# Identifying the performance and losses of a scroll compressor with vapour injection and R1234ze(E)

**DOI:** 10.12688/openreseurope.14658.2

**Published:** 2022-11-28

**Authors:** George Meramveliotakis, George Kosmadakis, Sotirios Karellas

**Affiliations:** 1 Thermal Hydraulics and Multiphase Flow Laboratory, National Center for Scientific Research Demokritos, Agia Paraskevi, 15341, Greece; 2 Laboratory of Steam Boilers and Thermal Plants, National Technical University of Athens, Athens, 15780, Greece

**Keywords:** scroll compressor, HFO refrigerant, R1234ze(E), semi-empirical model, suction pressure drop, vapour injection, heat pump

## Abstract

This work investigates a vapour injection scroll compressor integrated in a heat pump using the refrigerant R1234ze(E). The water-to-water heat pump was tested under a wide temperature range at the evaporator and condenser sides. The test results revealed that the performance is significantly reduced for lifts of over 30 K with the coefficient of performance being even below 2 and the maximum 2 ^nd^ law efficiency was just 28%. In order to enlighten the reasons behind such significant compressor underperformance, a semi-empirical model has been extended to include vapour injection, and a new improved modelling approach for the suction pressure drop was developed and implemented considering both the turbulent and laminar inlet flow regimes. Once the accuracy of the developed semi-empirical model was verified, the model was then adjusted to account for the R1234ze(E) operation, by fine-tuning its parameters based on the test data. The main loss mechanism identified is the high suction pressure drop, due to the high friction factor, with the inlet refrigerant flow possibly being laminar instead of turbulent. This resulted in a significant reduction of the mass flow rate and volumetric efficiency, while the standard model for suction pressure drop was not able to capture this effect.

## Introduction

Hydrofluorocarbon (HFC) refrigerants are widely used in heat pumps and refrigeration systems. Although many of those have zero ozone depletion potential (ODP), their contribution to the global warming effect is still significant. According to the EU F-Gas regulation ^1^ and Kigali amendment to the Montreal Protocol ^2^, the use of HFCs is to be reduced, gradually moving towards their phase out due to the imposed strict limitations regarding their global warming potential (GWP). The most common HFCs such as R134a, R407C and R410A have a GWP of over 1400 ^3^ and will need to be replaced by refrigerants that combine both low GWP and acceptable performance in heat pump systems. For this purpose, many alternatives are examined and evaluated focusing on low GWP refrigerants preferably with a GWP less than 150 ^1^. Several refrigerants have been proposed such as natural refrigerants (ammonia and CO _2_), hydrocarbons (HCs), hydrofluoroolefins (HFOs), hydrochlorofluoroolefins (HCFOs) and mixtures of these substances ^4^.

HFOs are characterized by very low GWP (GWP<10), zero ODP, low or no flammability, non-toxicity ^5^ and can be considered as promising HFC replacements. The most widely used HFO is R1234yf, charged in many automotive air conditioning systems as a replacement of R134a ^6^. Another alternative to R134a for medium temperature applications is R1234ze(E) ^5, 7^ with a GWP equal to 4, which is applied as a pure substance or a mixture component in many applications ^8^. To evaluate its potential, Babiloni *et al.* ^9^ tested a vapour compression system with an internal heat exchanger (IHX) operating with R1234yf and R1234ze(E). Their results showed that the volumetric efficiency with both HFOs reduced by 3% to 6% compared with R134a, leading to a cooling capacity decrease of 9% and 30% respectively. In terms of performance, the coefficient of performance (COP) of R1234yf was 3 to 11% lower than that of R134a, with a similar reduction for R1234ze(E) (2% to 8%). Another study by Jankovic *et al.* ^10^ performed both experimental and numerical evaluation of R1234yf and R1234ze(E) as R134a replacements in a refrigeration system. Their overall conclusions are similar as the ones of Reference 9, since the cooling capacity was reduced by 6% and 27% with R1234yf and R1234ze(E) respectively, while the COP decrease was below 5% for both HFOs. In general, R1234yf was proposed as a more suitable replacement for low to medium temperature heat pumps, while R1234ze(E) for higher temperature purposes ^11^.

Mendoza *et al.* ^12^ extended the refrigerant range of R134a alternatives, by also investigating the HFO blend R450A, as well as R1234yf and R1234ze(E) in a reciprocating compressor. Their tests were conducted over a wide range of operating conditions, and they developed a model based on dimensionless numbers, in order to examine their performance. Higher volumetric and isentropic efficiencies were observed for R450A compared with the other alternative refrigerants, but still lower than those seen in R134a by about 5-6%. Moreover, R1234ze(E) had the lowest cooling capacity, but its COP was slightly lower than the one of R134a and higher than R1234yf and R450A.

It becomes clear that the main deficit of HFOs is the lower capacity. To overcome this, especially at high temperature lifts, vapour injection has been proposed that finds application in scroll and screw compressors ^13^. The main advantage of this technique is the rise of the capacity by controlling the injected refrigerant mass flow rate ^14^. Furthermore, it provides cooling to the compressor, decreasing the discharge temperature ^15^ and therefore, the operating envelope of the compressor can be expanded, compared to the standard vapour compression cycle. Additionally, the injection technique, especially with liquid refrigerant, decreases the oil temperature leading to better oil performance and reduces oil degradation effects ^16^. Lumkin *et al.* ^17^ generated dimensionless correlations to map the compressor performance based on the Buckingham-PI method using experimental data of a R407C scroll compressor with vapour and liquid-vapour injection. Also, the proposed correlations, were validated using data points from a variable-speed R410A vapour injection scroll compressor providing very high accuracy especially for predicting the injection ratio and compressor power.

Winandy and Lebrun ^18^, Cho *et al.* ^19^ and Wang *et al.* ^20^ have investigated the effect of vapour injection on heat pump systems with scroll compressors using HFCs, showing encouraging test results for heating capacity and COP. In the case of HFOs, Shuxue *et al.* ^21^ examined the performance of a vapour injected scroll compressor in a heat pump configuration with R1234yf and a R1234yf/R32 mixture and tested for low ambient temperatures. The heating capacity was improved due to the increase of the compressor discharge mass flow from 5% to 20% compared to the non-injected case. Shuxue and Guoyuan ^22^ performed similar tests on a vapour injection scroll compressor but with R1234ze(E) and a R32/R1234ze(E) mixture for electric vehicles air conditioning. The evaporating temperatures were low, and the operation with R1234ze(E) and vapour injection extended the minimum temperature limit to below -20 °C, and at the same time significantly increasing the heating capacity and the COP compared with the system without vapour injection.

Additionally to the experimental investigations of compressors with vapour injection, several relevant numerical studies have been also carried out. Fukuda *et al.* ^23^ evaluated R1234ze(E) and R1234ze(Z) as alternative fluids for high temperature heat pumps (HTHPs) which are classified based on the output temperature in the range between 100 – 160 °C ^4^. The main results revealed major inadequacies between theoretical and experimental findings, mostly related to the COP values, due to significant reduction of the volumetric capacity caused by irreversible losses. Further analysis of the losses showed that R1234ze(Z) provided better performance at high temperatures, as also concluded in Reference 24, because the losses related to the pressure drop were significantly reduced. For that reason, these two refrigerants were proposed as suitable ones for HTHPs. Regarding other numerical models, Wang *et al.* ^25^ developed a geometrical model for a scroll compressor with vapour injection operating with R22. Based on simulations and experiments, the refrigerant injection was modelled as a parameter-varying process described by adiabatic throttling of the injected refrigerant up to the pressure inside the scroll pocket, with a simultaneous isobaric mixing of the two streams. Another geometrical model was developed in Reference 26 for a variable speed scroll compressor by using continuity, energy conservation and real gas equations. The model was validated with R22 and the injection conditions as well as the port geometry were analyzed based on the rotational frequency.

Due to the high complexity and computational cost of the geometrical models, semi-empirical models have also been developed to describe the several processes within the compressor. Winandy and Lebrun ^18^ developed such model for both vapour and liquid injection with its parameters fine-tuned for R22. The injection was assumed to be carried out after the closure of the suction pocket while leakages and pressure drops were not considered. The prediction accuracy was within ±4%, ±4.5%, and ±5 K for the mass flow rate, compressor power and discharge temperature of the compressor respectively. Another semi-empirical model was developed by Dardenne *et al.* ^27^ for a variable speed scroll compressor with R410A. The vapour injection is described by decomposing the process into several steps during the isentropic part of the compression. The injection flow in the compressor is not considered constant, ^28^, but rather it varies according to the pressure difference between the injection line and the compressor pocket. The semi-empirical model also includes compressor leakages, resulting in a set of 10 parameters that need to be fine-tuned with experimental data. A different approach has been followed by Tello-Oquendo *et al.* ^29^, who used a linear correlation of the injected mass flow as a function of the intermediate pressure and the given suction conditions. The model takes into consideration the main sources of losses such as the suction and discharge pressure drop and the compressor leakages. For the model validation, different non-injected scroll compressors were tested with R290 and a vapour injection scroll compressor with R407C. Their results revealed that for the latter the isentropic and volumetric efficiency was predicted with a deviation lower than ±5% compared with the experimental data.

Overall, the low GWP HFO refrigerants, such as R1234yf and R1234ze(E), provide a decreased performance and capacity, when they are used as drop-in alternatives. In order to combat this effect, vapour injection has been proposed in scroll compressors as a promising way to enhance the system capacity. But still the loss mechanisms are not well defined compared with the ones using the standard HFC refrigerants, with the relevant experimental facilities aiming mostly to identify the overall performance and capacity. The current work aims to cover this gap, by following a combined experimental and numerical approach. Once the semi-empirical model is developed with advanced features, including a more precise model for the suction pressure drop, and fine-tuned with the use of the test data, it is then applied to provide a better understanding of the compressor losses with the final objective to locate the reasons for low heat pump performance and 2 ^nd^ law efficiency.

## Improving the semi-empirical model

The semi-empirical model has been verified in Reference 29 for different scroll compressors (without vapour injection) operating with a variety of HFC and HFO refrigerants relying on the initial methodology proposed by Winandy *et al.* ^30^. The model is further extended here to describe vapour injection, in order to evaluate the prediction accuracy and compressor performance based on experimental results with R1234ze(E) refrigerant. At the same time, a new numerical approach for modeling the suction pressure drop has been developed, considering both laminar and turbulent flow at the suction line, aiming to further improve the prediction accuracy, even at conditions outside the operating envelope of the compressor.

### Semi-empirical model of a scroll compressor with vapour injection

The model introduces one injection line at the compressor and splits the compression into two stages. The first one refers to the isentropic compression from the suction conditions to the intermediate state, while the second one considers the refrigerant after the adiabatic mixing up to the adapted conditions (identified by the built-in volume ratio). The intermediate state is where the vapour is injected, and its properties are identified via corresponding pressure, temperature, and mass flow rate. The refrigerant properties are identified through the built-in thermodynamic libraries available in the Engineering Equation Solver (EES) software ^31^. As an open-source alternative, many thermodynamic libraries are also available and can be adapted in programing languages like Python. The complete evolution of the refrigerant in the compressor with the different processes that take place are described next.

1. Isobaric heating-up of the supply (su→su1), due to the hot motor and the casing. This process is related to the operating pressure ratio ^32^ and affects the actual trapped volume of the refrigerant, having a major impact on the volumetric efficiency of the compressor.
2. Adiabatic pressure drop at the suction port (su1→su2) which further influences the inlet conditions, by reducing the refrigerant pressure and density. Once this pressure drop increases, less refrigerant mass enters the compression chamber, leading to a reduction of the volumetric efficiency and as a result, of the overall performance.
3. Adiabatic mixing of supply flow with the leakage flow (su2→su3) due to the clearances between the bottom and top plate as well as the sidewalls of the scrolls.
4. Isentropic compression of the primary suction mass flow up to the intermediate pressure (su3→int), in which the vapour injection takes place. The intermediate state is identified by assuming the same absolute pressure between the injection suction line and the position inside the compressor after the injection port.
5. Isobaric mixing of the compressed suction mass flow with the injected vapour (int→int1).
6. Isentropic compression of the mixed flow up to the adapted conditions (int1→ad) which correspond to the built-in volume ratio of the compressor.
7. Adiabatic compression at constant volume due to the opening of the discharge valve from the adapted state up to the exhaust pressure (ad→ex1).
8. Leaking flow to the compressor inlet (ex1→ex1.1) due to the pressure difference that pushes out refrigerant from the clearances. The internal leakages are modelled assuming isentropic flow through a convergent nozzle with a cross-sectional area of the throat equal to the total leakage area of the compressor.
9. Isobaric cooling down at a constant wall temperature of T _w_ (ex1→ex2) due to the heat transfer between the high-temperature discharge refrigerant and the compressor casing. A part of this heat is lost to the ambient through the casing.
10. Adiabatic pressure drop at the exhaust port to the final discharge pressure (ex2→ex). This pressure loss is modelled assuming isentropic flow in a converging nozzle followed by an isobaric diffuser.

**Figure 1.**
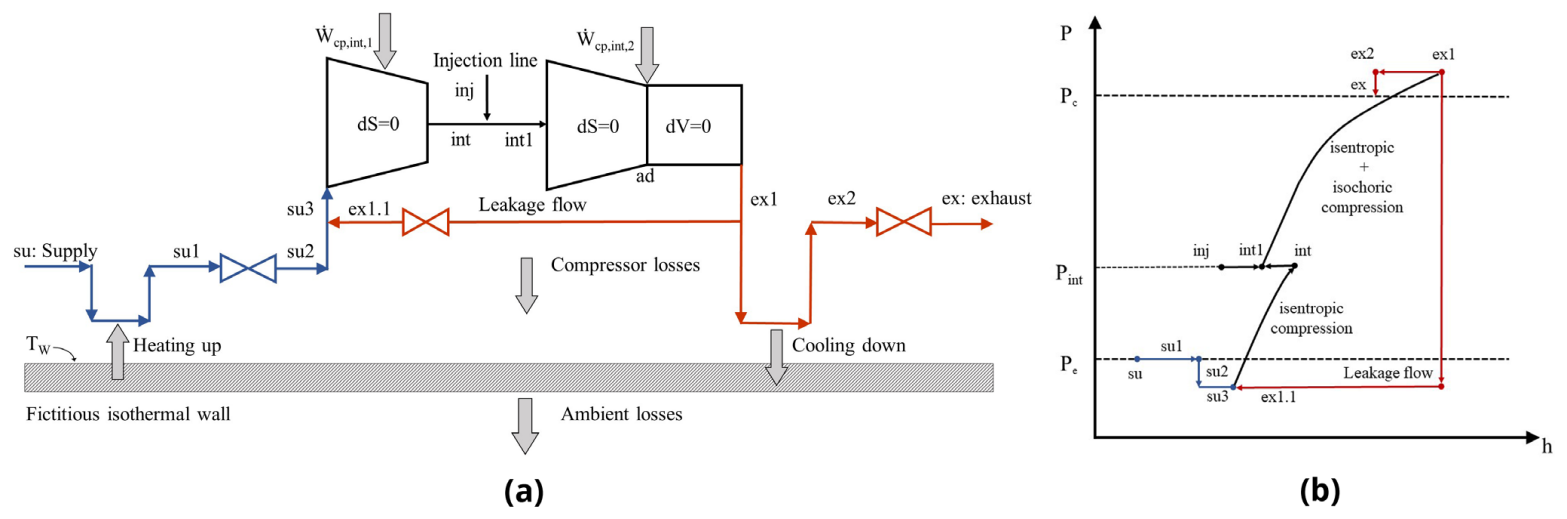
Compressor sub-processes with vapour injection (su1: supply after heating up; su2: supply after heating up and pressure drop; su3: inlet to compressor; dS=0: isentropic; -pcp,int,1: power of 1 ^st^ stage compression; int: outlet of 1 ^st^ compression stage; inj: injection; int1: inlet to 2 ^nd^ compression stage; etcp,int,2: power of 2 ^nd^ stage compression; ad: adiabatic; dV=0: isochoric; ex1: outlet of 2 ^nd^ compression stage; ex2: outlet after cooling down): ( **a**) schematic diagram highlighting the different states; ( **b**) generic pressure-enthalpy chart.

The compressor sub-processes including vapour injection and the corresponding pressure-enthalpy diagram are shown in Figure 1, with the supply side highlighted in blue, the compression with black and the exhaust with red colored lines. Further details of each sub-process along with their mathematical formulation are found in Reference 29.

The input data are the compressor supply temperature (T _su_), the evaporating and condensing pressures (P _su_ and P _ex_), the ambient temperature (T _amb_), the compressor speed (N _cp_), and the ones related to the vapour injection: the intermediate pressure and temperature (P _int_ and T _int_) and the injected mass flow rate (ṁ _vi_).

The sub-processes highlighted above introduced a set of 12 parameters in total, that are fine-tuned with the use of available data from either the free software of the compressor manufacturer or experimental results, as discussed later in this work. This is achieved once minimizing the defined function Θ ( Equation. (1)) by implementing a genetic algorithm optimization process ^33^.


$$\Theta \text{=}\sqrt{\frac{1}{\text{Z}}\sum_{\text{ι}=1}^{\text{Z}}\left[{\left(\frac{{\text{T}}_{\text{calc,i}}\text{-}{\text{T}}_{\text{data,i}}}{{\text{T}}_{\text{data,i}}}\right)}^{2}+{\left(\frac{{\dot{\text{m}}}_{\text{calc,i}}\text{-}{\dot{\text{m}}}_{\text{data,i}}}{{\dot{\text{m}}}_{\text{data,i}}}\right)}^{2}+{\left(\frac{{\dot{W}}_{\text{calc,i}}\text{-}{\dot{W}}_{\text{data,i}}}{{\dot{W}}_{\text{data,i}}}\right)}^{2}\right]}\;(1)$$

Where Z is the total number of datasets, subscripts *calc* and *data* correspond to the calculated values and the available data and T, ṁ and *Ẇ* refer to the discharge temperature, mass flow rate and electric power respectively.

**Figure 2.**
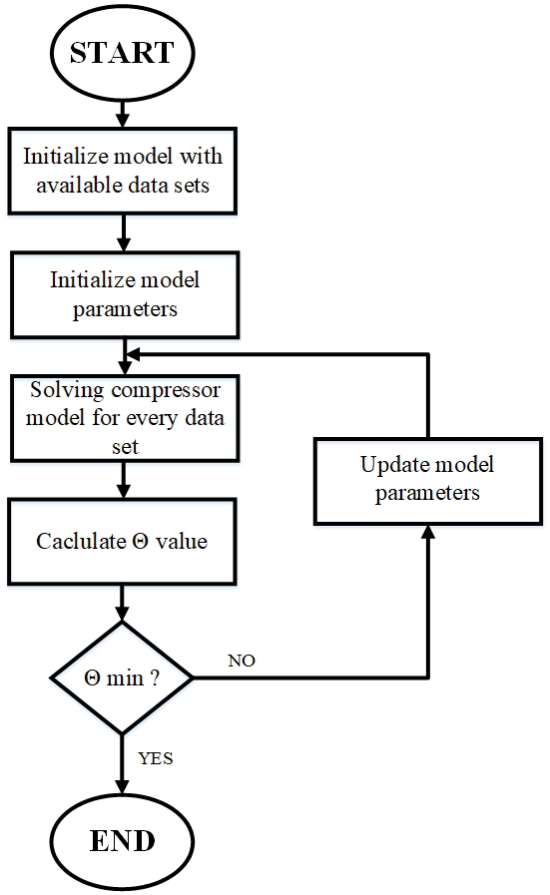
Flowchart of the compressor model.

The basic compressor model algorithm is depicted in Figure 2 and was developed under the EES environment ^31^ due to the flexibility of thermodynamic properties calculations and built-in optimization tools, selecting the genetic algorithms for this work to reach the global optimum.

The set of the model parameters is shown in Table 1. The parameters D _ex_ and corr are identified according to technical specifications given by the compressor manufacturer and the literature respectively, while ṁ _r,cp,n_ is the reference mass flow rate calculated from the nominal swept volume multiplied by the density for a saturated vapour at 0 °C. The other nine parameters are identified by the function Θ minimization.

**Table 1.** Parameters of the semi-empirical model and relevant compressor sub-processes with vapour injection.

Parameter	Units	Sub-process
*AU _su,cp,n_*	W/K	Heat transfer coefficient for heating-up during supply
*AU _ex,cp,n_*	W/K	Heat transfer coefficient for heat loss during the exhaust
*AU _amb_*	W/K	Heat transfer coefficient for heat losses to the ambient
*A _leak_*	m ^2^	Effective area of leakages
*Ẇ _cp,loss,0_*	W	Constant term of the electrical power losses
*α _cp_*	-	Coefficient of the variable term of the electrical power losses
*D _ex_*	m	Exhaust diameter for discharge pressure losses
*corr*	1/W	Motor slip coefficient
*r _v,in_*	-	Built-in volume ratio of the compression process
*ṁ _r,cp,n_*	kg/s	Nominal mass flow of the refrigerant
*V _s, cp_*	m ^3^	Swept volume of the compressor
*Κ*	1/m ^4^	Lumped friction factor parameter

In order to model the whole vapor injection process, two more equations are needed related to steps 4, 5 and 6 presented previously. The isobaric mixing between the injection flow and the compressed mass inside the scrolls are described by Equation. (2) and Equation. (3) respectively.


$$\;{\dot{\text{m}}}_{\text{vi}}{\text{h}}_{\text{r,inj}}\text{+}{\dot{\text{m}}}_{\text{r,cp,su3}}{\text{h}}_{\text{r,int}}\text{=}{\dot{\text{m}}}_{\text{r,cp}}{\text{h}}_{\text{r,int1}}\;(2)$$

$$\;{\dot{\text{m}}}_{\text{r,cp}}\text{=}{\dot{\text{m}}}_{\text{vi}}\text{+}{\dot{\text{m}}}_{\text{r,cp,su3}}\;(3)$$

$$\;{\text{R}}_{\text{vi}}=\frac{{\dot{\text{m}}}_{\text{vi}}}{{\dot{\text{m}}}_{\text{r,cp,su3}}}\;(4)$$

Where ṁ _vi_ is the injected mass flow rate, ṁ _r,cp,su3_ is the mass flow rate after the first compression stage, ṁ _r,cp_ is the compressor mass flow after mixing, h _r,inj_ is the refrigerant enthalpy at the injection line, h _r,int_ is the refrigerant enthalpy at the intermediate state inside the compressor, h _r,int1_ is the enthalpy after the mixing of the two streams, and R _vi_ is the ratio between the injected and suction mass flow rates.

Due to the splitting of the whole compression into two stages, the calculation of the compression work requires an additional term. The total internal work provided to the compressor (w _in_) is then given by Equation. (5).


$${\text{w}}_{\text{in}}{\text{=}\;\text{w}}_{\text{in1,is}}\text{+}{\text{w}}_{\text{in2,is}}{\text{+w}\;}_{\text{in,v}}{\text{=}(\text{h}}_{\text{r,int}}\text{-}\;{\text{h}}_{\text{r,su3}}{\text{)}+\text{(h}}_{\text{r,ad}}{\text{-h}}_{\text{r,int1}})\text{+}{\text{v}}_{\text{r,ad}}({\text{P}}_{\text{ex1}}{\text{-P}}_{\text{ad}}\text{)}\;(5)$$

Where w _in1,is_ and w _in2,is_ is the required work for the isentropic compression from the initial (su3) to the intermediate (int) and from the intermediate to the built-in (ad) volume respectively, the subscript r refers to the refrigerant side and the int1 represents the thermodynamic state obtained after the isobaric mixing with the vapour injection line. Also, w _in,v_ is the work corresponding to the constant volume part and is obtained by the constant specific volume calculated in the adapted conditions (v _r,in_) multiplied by the pressure difference of the exhaust (P _ex1_) with the adapted (P _ad_) state.

The main performance indicators of the compressor are the volumetric and isentropic efficiency determined by Equation. (6)– Equation. (7). The volumetric efficiency (η _vol_) is defined as the ratio of the actual mass flow that enters the compressor to the theoretical one, calculated at the suction position by using the nominal volumetric flow rate ^34^. The isentropic efficiency (η _is_) expresses the ratio of the ideal to the actual power consumption, adjusted for vapour injection compressors, as proposed in References 17, 35 according to ASHRAE (American Society of Heating, Refrigerating and Air-Conditioning Engineers) standard 23.1 ^36^.


$$\;{\text{n}}_{\text{vol}}\text{=}\frac{{\dot{\text{m}}}_{\text{r,cp,su3}}}{{\text{ρ}}_{\text{su}}{\dot{\text{V}}}_{\text{cp}}}\;(6)$$

$$\;{\text{η}}_{\text{is}}\text{=}\frac{{\dot{\text{m}}}_{\text{r,cp,su3}}({\text{h}}_{\text{r,int,is}}\text{-}{\text{h}}_{\text{r,su3}})+{\dot{\text{m}}}_{r,cp}({\text{h}}_{\text{r,ex,is}}\text{-}{\text{h}}_{\text{int1}})}{{\dot{\text{W}}}_{\text{cp}}}\;(7)$$

Where ρ _su_ is the density at the compressor suction, $\dot{\text{V}}$ _cp_ is the volumetric flow rate calculated by the nominal swept volume and the rotational speed ^37^, h _r,int,is_ is the outlet enthalpy once the refrigerant is compressed isentropically from the suction up to the intermediate state, h _r,ex,is_ refers to the specific enthalpy of the fluid following an isentropic compression from the intermediate state after mixing with injection line up to the discharge, and Ẇ _cp_ is the total electrical power provided to the compressor that includes the electromechanical conversion and all related losses.

### Development of an improved suction pressure drop model

The numerical model of the suction pressure drop that has been previously included in the compressor model is the one proposed by Tello-Oquendo *et al.* ^29^. This is based on the Darcy-Weisbach equation introducing a non-dimensionless friction factor that includes all the geometric characteristics that affect pressure drop ( Equation. (8)).


$$\text{Δ}{\text{P}}_{\text{suc}}\text{=f}\cdot \rho_{su}\cdot \frac{{\text{u}}^{2}}{2}\text{=f⋅}\rho_{su}\cdot \frac{{\left({\text{n}}_{\text{vol}}{\dot{\text{V}}}_{\text{s}}\right)}^{2}}{2\text{⋅}{\text{A}}_{\text{su}}^{\text{2}}}\text{=K}\cdot \frac{\rho_{su}}{2}\cdot {\left({\text{n}}_{\text{vol}}{\dot{\text{V}}}_{\text{cp}}\right)}^{2}\;(8)$$

Where f is the Darcy-Weisbach friction factor, *u* is the inlet velocity, A _su_ is the effective area of the suction port, and *K* is the lumped friction factor parameter that includes all the geometric features.

When the compressor operates within its envelope, the flow at the suction is typically a fully developed turbulent one, resulting to an almost constant friction factor according to the Moody chart ^38^. However, when the compressor operation exceeds the envelope limits or the properties of the working fluid differ from the ones of the compressor’s compatible refrigerants, the fluid flow may not remain turbulent. In the case of a low refrigerant suction pressure and density, the flow velocity is reduced, possibly reaching the laminar regime, in which the friction losses are proportional to the velocity. The outcome is a strong variation of the friction factor with a Reynolds number that needs to be modelled with a suitable correlation.

Such correlation has been developed in this study, by initially examining the effect of the flow regime on the friction factor. In the case of a laminar flow (with *Re<2300*) the Hagen-Poiseuille equation ^39^ ( Equation. (9)) applies.


f=64Re⁡(9)

Where *Re* is the Reynolds number of the working fluid.

For a transient or turbulent flow ( *Re>2300*), the friction factor is commonly described by the Colebrook-White equation ^40^ ( Equation. (10)).


f=-2log10(2.51Re⁡f+ε3.71D)(10)

Where *ε/D* is the relative roughness of the inner surface of the inlet pipe, with its range of practical interest between 10 ^-6^ and 0.05.

Several correlations have been developed that provide the friction factor over an extended Reynolds number range with a good accuracy ^39^. Some common ones for both laminar and turbulent regimes and all relative roughness ranges are given in Table 2. These expressions are implicit functions including the relative roughness and the *Re*.

**Table 2.** Literature correlations for Darcy-Weisbach friction factor calculation.

Correlations	Reference
$\text{f}=5.5\cdot 10^{3}\left[1+{\left(2\cdot 10^{4}\left(\frac{\varepsilon }{\text{D}}\right)+\frac{10^{6}}{\text{Re}}\right)}^{\frac{1}{3}}\right]$	Moody (1947) ^38^
$\text{f}=-2\;{\text{log}}_{10}\left[\left(\frac{\frac{\varepsilon }{\text{D}}}{3.7065}\right)-\left(5.0452\cdot \frac{\text{A}}{\text{Re}}\right)\right]$ where: $\text{A}={\text{log}}_{10}\left[\frac{{\left(\frac{\varepsilon }{\text{D}}\right)}^{1.1098}}{2.8257}+\frac{5.8506}{{\text{Re}}^{0.8981}}\right]$	Chen (1979) ^41^
$\text{f}=-2\;{\text{log}}_{10}\left[\left(\frac{\text{ε}/\text{D}}{3.7}\right)-\frac{5.0.2\cdot \text{B}}{\text{Re}}\right]$ where: $\text{A}={\text{log}}_{10}\left[\frac{\text{ε}/\text{D}}{3.7}+\frac{13}{\text{Re}}\right],\;\text{B}={\text{log}}_{10}\left[\frac{\text{ε}/\text{D}}{3.7}-\frac{5.02\cdot \text{A}}{\text{Re}}\right]$	Zigrang & Sylvester (1982) ^42^
$\text{f}=-2\;{\text{log}}_{10}\left[\left(\frac{\frac{\varepsilon }{\text{D}}}{3.7065}\right)-\left(5.0272\cdot \frac{\text{B}}{\text{Re}}\right)\right]$ where: B=log⁡10[εD3.827-(4.567ARe⁡)], $\text{A}={\text{log}}_{10}\left[{\left(\frac{\frac{\varepsilon }{D}}{7.7918}\right)}^{0.9924}+\left(\frac{5.3326}{208.815+Re}\right)\right]$	Monzon-Romeo-Royo (2002) ^43^

With the aim to expand the validity of the calculations over the whole range of the Reynolds number, and at the same time to avoid the estimation of the unknown geometric characteristics, a logarithmic expression is developed as a function of only the Reynolds number, instead of using an implicit equation from the available ones. The necessity of a new equation is based on the fact that the $\frac{\text{ε}}{\text{D}}$ ratio at the compressor inlet is an unknown parameter. For that reason, all the geometrical parameters that impact on the friction factor are lumped in the calibration parameters A _0_ and A _1_ of Equation. (11).


f(Re)=A0+A1[log⁡10(Re⁡)]-3(11)

This type has been preferred due to the logarithmic relation between friction factor and Reynolds number as shown in the relevant literature ^39^. This equation involves two parameters that need to be calibrated, in order for its results to match with the ones of Table 2 for every $\frac{\text{ε}}{\text{D}}$ value. The inverse cubed exponent in Equation. (10) is chosen by a best-fit procedure in order for the correlation to closely follow the results of the literature expressions over the entire range of Reynolds number. The fitting formula has been adjusted to the friction factor equations with a least squares method based on the Zigrang & Sylvester correlation ^42^.

**Figure 3.**
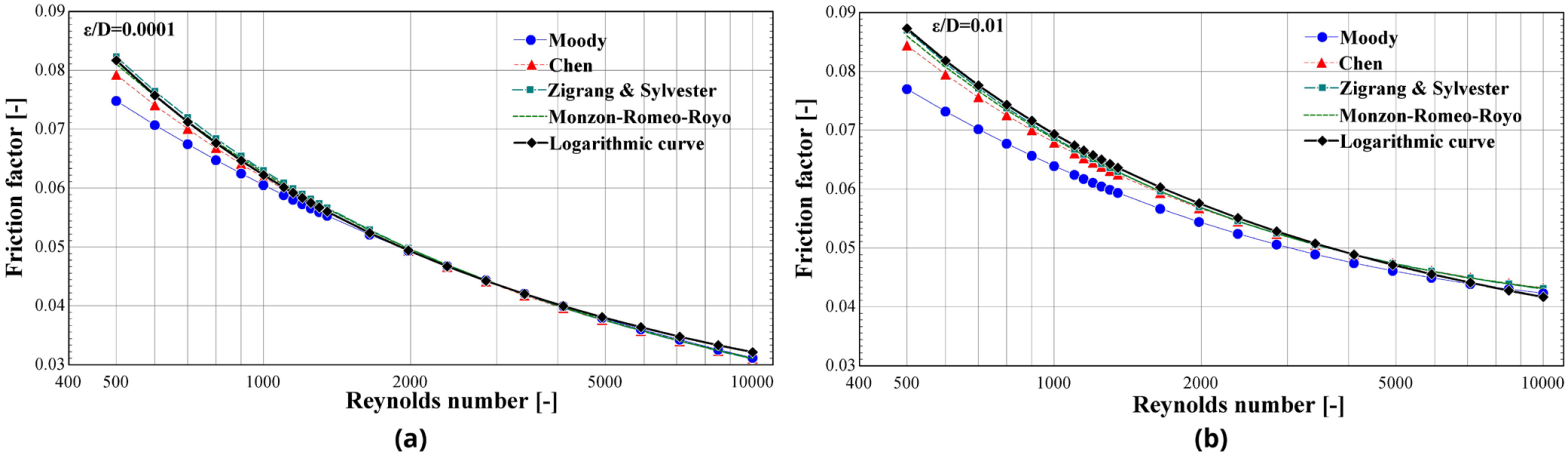
Friction factor as calculated by the proposed equation and the expressions of Table 2 over a large range of Reynolds numbers and for two ε/D ratios: ( **a**) 0.0001; and ( **b**) 0.01.

To demonstrate the accuracy of this expression, Equation. (11) is plotted in Figure 3 together with the expressions of Table 2 for a large range of Reynolds number (covering both laminar regimes with Re<2300 and turbulent ones) and for two extreme $\frac{\text{ε}}{\text{D}}$ ratios (0.0001 and 0.01) that bound the typical range in compressors for refrigeration and heat pump systems.

The proposed correlation closely follows the calculations of the four expressions. Moreover, it provides almost identical results with the Monzon-Romeo-Royo correlation ^43^ for both relative roughness values. It should be highlighted that this expression is the most recent from the available literature and includes all previous findings on this topic.

Finally, in order to integrate Equation. (11) in the suction pressure drop calculations, it is preferred not to use the Reynolds number as the independent variable, whose calculation includes the effective diameter of the suction port, which is an unknown. Instead, a similar parameter to the Reynolds number is used, which considers only the mass flow rate and the viscosity. This is achieved by multiplying the Reynolds number with the constant term $\left(\frac{\text{πD}}{4}\right)$ , as shown in Equation. (12). The final correlation of the friction factor is given by Equation. (13).


$$\;\text{X}\;\text{=}\frac{\text{π}\;\text{D}\;\text{Re}}{4}\text{=}\frac{{\dot{\text{m}}}_{\text{r,cp,su3}}}{\text{μ}}\;(12)$$

$$\;\text{f}(\text{X})\text{=}A_{0}^{\prime }\text{+}A_{1}^{\prime }\;{\left(\log_{10}(\text{X})\right)}^{\text{-}3}\;(13)$$

Where *μ* is the fluid viscosity, D is the suction port diameter and $A_{0}^{\prime }$ and $A_{1}^{\prime }$ are the adjusted parameters that include the geometrical values that impact on suction pressure drop and are introduced to the set of the model parameters to be determined by the Θ function minimization ( Table 1).

As a result, the initial constant friction factor is replaced with the expression of Equation. (13), introducing an additional parameter to the model, and leading to 13 parameters in total. The verification of this approach will be presented in a next section.

## Testing facilities of a vapour injection compressor

The tested vapour injection compressor is the Copeland ZH13KVE-TFD scroll compressor provided for R407C (GWP= 1980, ODP=0) with 11.7 m ^3^/h displacement at 50 Hz. This compressor is for heating applications and is equipped with an economizer port to allow the operation with vapour injection. The developed heat pump includes this compressor and the design work resulted in using R1234ze(E) with GWP =4 (20-years GWP) ^44^ and ODP=0 as drop-in replacement, expecting a heating COP reduction less than 10% compared with the same compressor running with R407C. The heat pump testing aimed to primarily examine and evaluate the reliability and performance of the compressor, with the heat pump to be later integrated in a prototype domestic heating application.

### Comparison of refrigerants R407C and R1234ze(E)

In order to overview the main characteristics of R1234ze(E) compared to R407C, the thermodynamic properties at 0 °C and 50 °C are shown in Table 3. The calculations have been performed within EES (Engineering Equation Solver) software ^31^, which includes integrated thermodynamic libraries of the examined refrigerants. R407C has higher latent heat and lower specific volume at both lower and higher temperatures, indicating a higher capacity and a more compact system.

**Table 3.** Thermo-physical properties of the two refrigerants.

	Thermo-physical properties									
Refrigerant	Latent heat (kJ kg ^-1^)		Specific Volume (m ^3^/kg)		Sat. liquid specific heat (kJ kg ^-1^K ^-1^)		Sat. vapor specific heat (kJ kg ^-1^K ^-1^)		Critical Temperature (°C)	Critical Pressure (Bar)
	0 °C	50 °C	0 °C	50 °C	0 °C	50 °C	0 °C	50 °C		
R407C	209.3	149.1	0.051	0.011	1.42	1.78	0.96	1.47	86.2	46.32
R1234ze(E)	184.2	145.1	0.084	0.018	1.34	1.51	0.90	1.15	109.4	36.32

**Figure 4.**
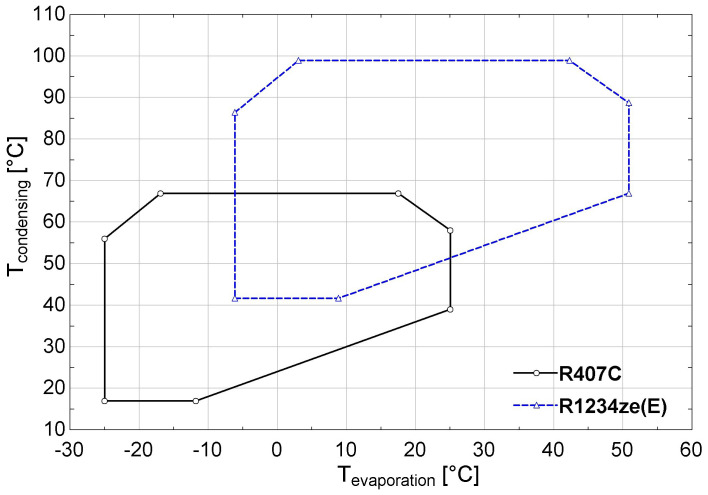
Compressor performance envelope of the Copeland ZH13KVE-TFD scroll compressor with R407C and R1234ze(E) refrigerant.

Concerning the operational envelope of the compressor, which is originally provided for R407C, this is transposed for R1234ze(E), by converting the compressor’s pressure ranges to the corresponding temperatures. The original for R407C and the adjusted for R1234ze(E) envelopes are shown in Figure 4. The heat pump with R1234ze(E) was tested near the lower limits of the envelope with lower suction pressure levels and reduced efficiency.

### Description of the heat pump test-rig

The experimental setup consists of a vapour compression heat pump for heating designed for R1234ze(E) and charged with a total refrigerant amount of 4 kg (supplied by Honeywell/Solstice® ZE). All three heat exchangers (HEX) are of plate type. Their sizing and selection was based on the manufacturer's software of SWEP taking into consideration both input-output temperatures and water mass flow rates provided by previous simulation results. Also, an over-surfacing of 25% has been applied in order to ensure that the performance of the HEX is kept always within the design conditions. As a result, model B25TH with 20 plates and 1.13 m ^2^ of heat exchange area has been selected for both condenser and evaporator, but with different couplings and dimensions for the inlet/outlet connections. For the economizer, model B5TH was selected, with 34 plates and a surface of 0.384 m ^2^.

**Figure 5.**
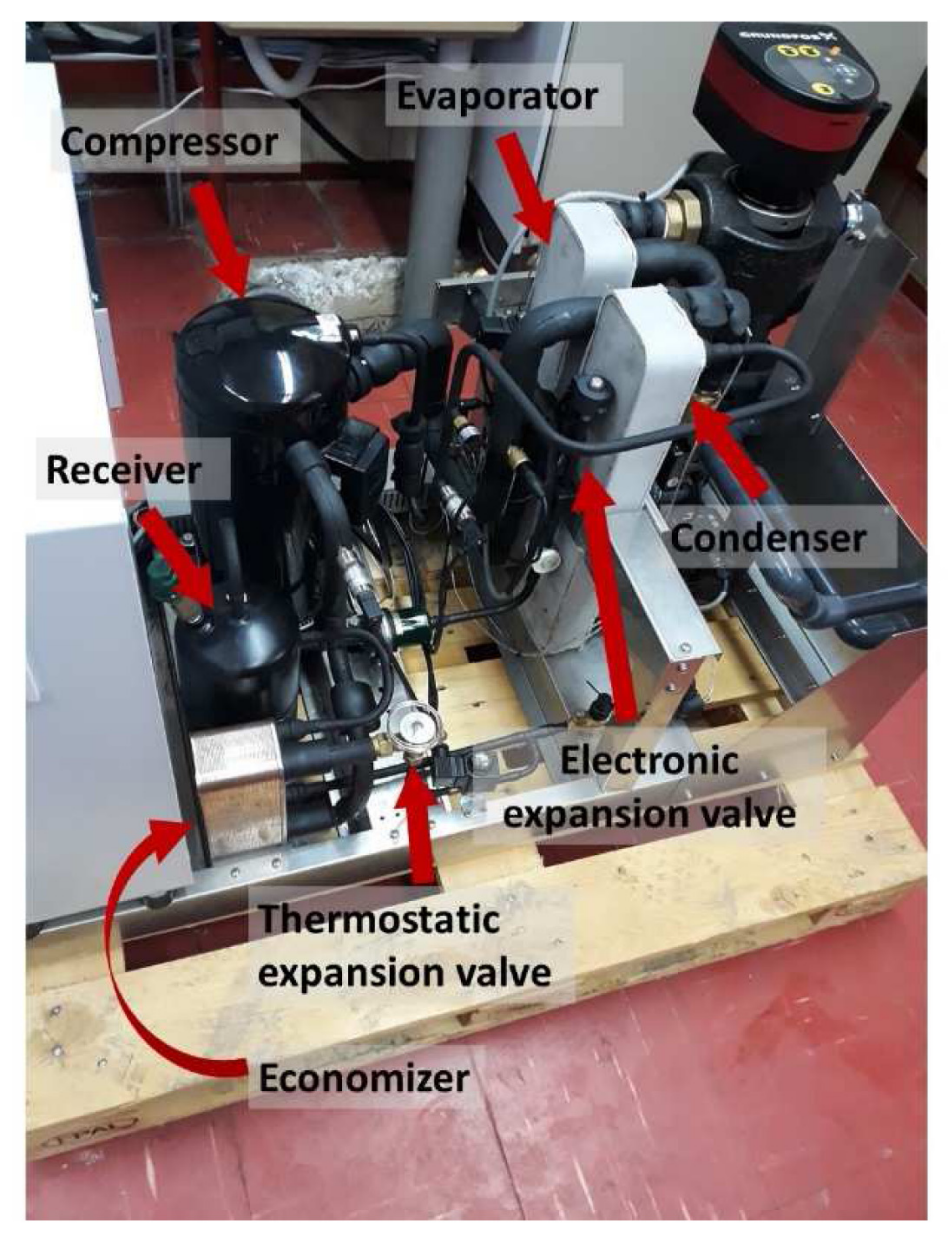
The heat pump tested at the laboratory.

The injection mass flow is determined by a thermostatic expansion valve placed at the condenser outlet, which provides some control of the superheat, keeping it within the range of 3–10 K. The selected expansion valve was the Danfoss T2/TE2 134 model and the control spring pressure was adjusted for the operation with R1234ze(E) by shifting the superheat compared to R134a according to the manufacturer. Concerning the superheat control at the compressor suction an electronic expansion valve (EEV) was preferred, and the one selected is produced by PARKER, type CEV24-S. The controller of this valve processes the measured temperature and pressure at the evaporator’s outlet and accordingly a series of electrical pulses are sent to the stator of the EEV causing to open or to close. The preferred control mechanism is a PID loop keeping the superheat in a very close range to the setpoint, equal to about 3 K. Moreover, a receiver with a volume of 1.5 l has been used produced by OCS (type RV1CC102X210) to account for changes in the operating conditions. After the receiver, a filter (GMC 5/8" SC165MM) is placed with a nominal volume of 250 cm ^3^. The developed heat pump installed at the laboratory during the tests is shown in Figure 5, indicating the main components.

The heat pump also includes two pumps for the water circulation between the heat exchangers and the water tanks. The water flow at the condenser is adjusted by controlling the pump speed with a low-voltage PWM (pulse-width modulation) signal. The maximum flow rate of the circulator pump is 4 m ^3^/h and head of 7 m with a power consumption below 60 W (Grundfos, type ALPHA1 L 15-65 130). For the evaporator water circuit, a booster pump has also been installed in series, ensuring that the flow rate is adequate. In this circuit, a circulator pump with permanent magnet motor (Grundfos, type MAGNA 3) is used with a maximum flow rate of 12 m ^3^/h and head of 10 m, delivering higher mass flow rates that can achieve lower water temperature differences.

**Figure 6.**
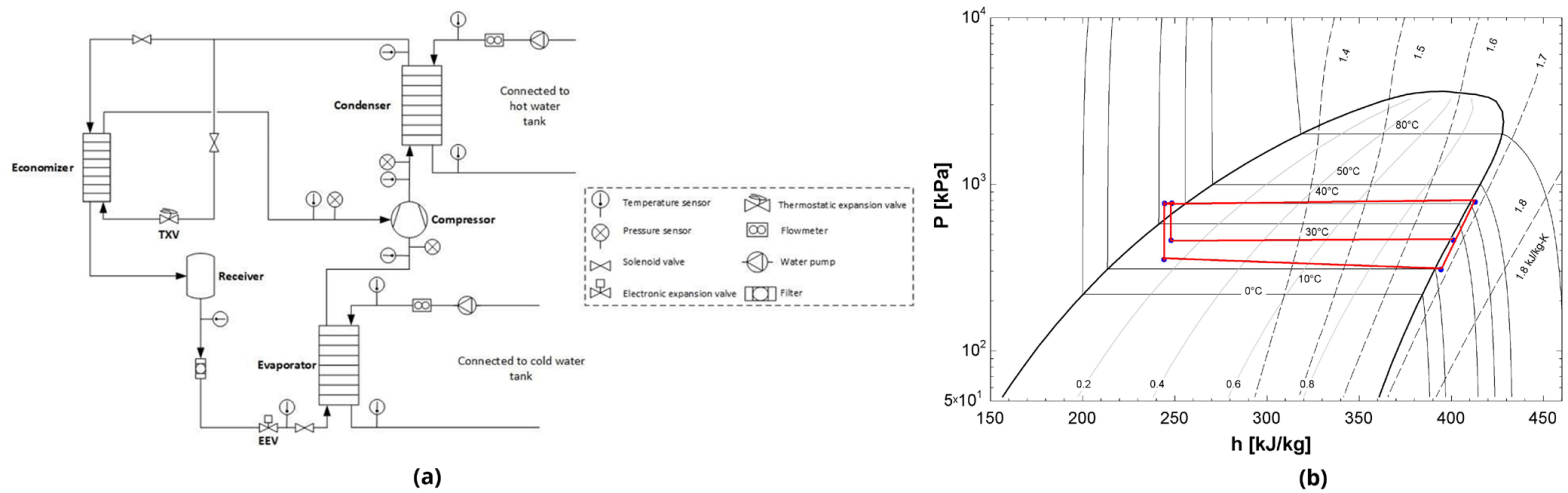
( **a**) Schematic diagram of the tested heat pump with measurement points, ( **b**) The corresponding P-h diagram.

The secondary circuits for the hot and cold side of the heat pump are connected to a different insulated water tank with a volume of 5 m ^3^ each. Concerning the tank temperature control, the evaporator water tank is equipped with electric resistors for heating up the stored water. On the other hand, the water tank connected to the condenser is equipped with an air-source heat pump to cool the water in the tank. By doing so, the inlet water temperatures to the heat pump can be stabilized at both evaporator and condenser, allowing for test to be performed with constant supply conditions. For a better overview, the piping and instrumentation diagram of the vapour injection heat pump is depicted in Figure 6, showing both the components and the measurement points.

### Sensors and their accuracy

Various sensors are installed for measuring the pressure of the refrigerant and the temperature of both the refrigerant and water at different parts of the cycle. In total, there are three pressure sensors and 10 temperature sensors (six for the refrigerant and four for the water lines). All measurements related to the refrigerant are collected and recorded by the Programmable logic controller (PLC) unit of the heat pump with a sampling rate of one minute.

Moreover, a power meter (Siemens, type SICAM T 7KG9661) measures the total electrical consumption of the heat pump. The measurements that are collected at the water side as well as the (indoor) ambient temperature and the total power consumption is recorded by a data logger (Agilent, type 34972A) with a sampling rate once every 30 seconds. These are then synchronized with the ones recorded by the PLC unit of the heat pump.

The sensors of the experimental setup together with their range and accuracy are presented in Table 4, given separately for the heat pump (by the PLC) and the test-rig sides (by the data logger).

**Table 4.** Accuracy and range of heat pump sensors and meters.

Meter/Sensor	Quantity	Type	Range	Accuracy
Test-rig				
Water flow meter (condenser)	1	Yokogawa ADMAG AFX electromagnetic flow meter	Adjustable (0–20,000 lt/h)	<1%
Water flow meter (evaporator)	1	Krohne IFC 010D electromagnetic flow meter	Adjustable (1–15,000 lt/h)	<1%
Water temperature	4	PT100, 4-wire	0–100 °C	0.1 K
Power meter	1	Siemens Sicam-T	0–50 kW per phase (with current transformers)	0.5%
Heat pump				
Refrigerant temperature	6	PT100, 3-wire	-20 – +80 °C	0.1 K
Refrigerant gauge pressure	3	Pressure transmitter ESCP- MIT1	0–10 bar for the evaporator and economizer lines 0–30 bar for the condenser line	0.25% of FS
Water temperature	4	PT100, 3-wire	0 – 100 °C	0.1 K

### Test conditions

All tests have been conducted with the evaporator inlet temperature ranging from 10 up to 25 °C while the water outlet of the condenser ranged from 32 to 55 °C. The tests were performed at constant water flow rates (1.35 m ^3^/h at the condenser and 2.18 m ^3^/h at the evaporator) and with a constant compressor frequency of 50 Hz. Three main testing sets have been obtained regarding the temperature range of the evaporator and condenser, by keeping the evaporator water inlet temperature at three temperature scales 10, 15 and 25 °C respectively, while the condenser water outlet is varied from 32 to about 55 °C with a constant elevating trend, since the heat pump was delivering heat to a large tank.

At the beginning of every testing set, the heat pump was operating to bring the temperature of the evaporator water tank to the desired level with simultaneous operation of the outdoor air-source heat pump, in order to reduce the water temperature of the condenser tank. As the water temperature of the evaporator’s tank was stabilized by adjusting the operation of the electric resistances, the water temperature of the condenser tank started to increase until it reached the maximum set-point of 55 °C.

**Figure 7.**
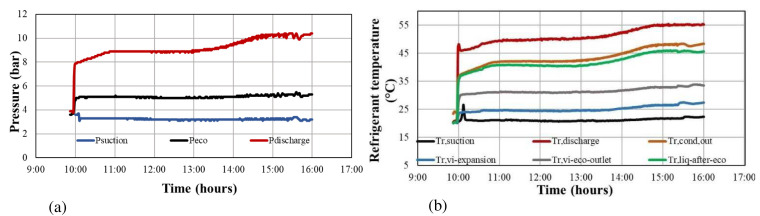
Sample of the refrigerant-side measured data for a six-hour heat pump operation: ( **a**) refrigerant pressures; ( **b**) refrigerant temperatures.

The heat pump was operating many hours per day (six to eight) only for experimental reasons and for consecutive days (about 3 weeks), recording all measured data and controlling the temperatures of the tanks. As a sample, Figure 7 shows the refrigerant temperatures and pressures during six hours of testing, when the water at the evaporator tank was maintained at about 25 °C while the condenser tank was heated from 45 up to 55 °C.

All collected measurements have been synchronised with a sampling rate of 1 minute, and then they were time-averaged into 10-minute steps. The final dataset is determined by selecting several such 10-minute sets with the measured values having small fluctuations during this averaging process. A small fluctuation is defined here as 1-minute measurements with a deviation of less than 5% compared to the 10-minute averaged value in terms of temperatures and pressures. Table 5 summarizes the range of the final measurement points that are taken into consideration, in order to evaluate the compressor and the overall heat pump performance.

**Table 5.** Main test conditions of the heat pump.

Property	Value
Refrigerant suction pressure (relative) (bar)	1.7 – 3.5
Refrigerant discharge pressure (relative) (bar)	6.24 – 11.6
Water inlet temperature to evaporator (°C)	9.82 – 26.66
Water outlet temperature from condenser (°C)	31.55 – 55.01
Total power consumption (kW)	2.23 – 3.09

### Data reduction

The key performance indicators and energy flows are calculated from the measured temperatures, pressures, flow rates and power consumption. The heating capacity (Q _c_) is identified from the water-side of the condenser as in Reference 45 given by Equation. (14), assuming no heat losses in order to enable the calculation of the refrigerant mass flow rate (ṁ _r,c_). Due to the lack of mass flow meters at the refrigerant side of the test rig, it is not possible to carry out confirmation tests for the heat rate balance on the water and refrigerant side respectively. However, the heat exchangers are sufficiently insulated and as a result it is assumed that no heat is lost in the HEX.


$$\;{\text{Q}}_{\text{c}}\text{=}{\dot{\text{m}}}_{\text{w,c}}\text{⋅}{\text{c}}_{\text{p,w,c}}\text{⋅}{\text{ΔT}}_{\text{w,c}}\text{=}{\dot{\text{m}}}_{\text{r,c}}\text{⋅}({\text{h}}_{\text{r,c,in}}{\text{-h}}_{\text{r,c,out}})\;(14)$$

where ṁ _w,c_ is the measured water mass flow rate, c _p,w_ is the specific heat capacity, and ΔT _w_ is the water temperature difference between the outlet and inlet port of the plate heat exchanger.

The total mass flow rate at the condenser, which is the discharge mass flow of the compressor, is calculated by an energy balance at the water side (first part of Equation. (14)). The refrigerant enthalpies at the condenser’s outlet (h _r,c,out_) and inlet (h _r,c,in_) are calculated by the measured pressures and temperatures. Τhe heating COP is calculated by Equation. (15). 


$$\;\text{COP}\;\text{=}\frac{{\text{Q}}_{\text{c}}}{{\dot{\text{W}}}_{\text{cp}}}\;(15)$$

where Ẇ _cp_ is the compressor electric power, which is equal to the electric power measured by the power meter including all power consumptions once reduced by the power consumption of all secondary components such as pumps, controllers etc. 

Except from COP, the 2 ^nd^ law efficiency is also be used as an indicator of how much of the theoretical Carnot (maximum) efficiency is achieved. According to Reference 46 the 2 ^nd^ law efficiency (η _II_, also called Carnot efficiency) is defined as the ratio of the required minimum energy input for an ideal system, to the actual energy input of the real system, given by Equation. (16).


$$\;\eta_{II}\text{=}\frac{COP}{COP_{ideal}}\;(16)$$

where COP _ideal _is the COP of the Carnot cycle which is related only with the temperature levels of the heat pump supply and sink (water) side.

The performance indicators of the compressor are the volumetric and isentropic efficiency identified by Equation. (6) and Equation (7) as it was proposed in a characterization methodology of a vapour injection scroll compressor ^34^. In Equation. (6), the refrigerant mass flow rate is identified at this time by applying an energy balance at the evaporator, similar to Εquation. (13). Moreover, by using the continuity equation ( Equation. (3)) and once the evaporator and condenser mass flow rates are determined, the vapour injection mass flow is then calculated. This enables the identification of the isentropic efficiency with the use of Equation. (7).

This processing method leads to the calculation of the key performance indicators. The selected operating conditions include 13 datasets. These have been obtained from the collected ones directly from the measurement points and then the heat losses and pressure drop in the piping have been considered, in order to form the final inlet and outlet conditions of each component. Further the data were screened and selected based on the minimum fluctuations of the measured values over the 10-minute period. The measured data of the heat pump are provided in Table 6, and will be used for the semi-empirical model calibration in the next section.

**Table 6.** Final measured dataset of the heat pump.

Ambient temperature (°C)	Suction temperature (°C)	Discharge temperature (°C)	Condenser outlet temperature (°C)	Receiver Liquid temperature (°C)	Vapour injection temperature (°C)	Suction pressure (bar)	Vapour injection pressure (bar)	Discharge pressure (bar)	Compressor power (W)
23.5±0.1	19.0±0.1	38.8±0.1	28.1±0.1	28.3±0.1	26.7±0.1	4.00±0.02	5.60±0.02	7.24±0.07	2292.00±11.46
20.8±0.1	19.2±0.1	44.5±0.1	35.7±0.1	34.8±0.1	28.3±0.1	4.00±0.02	5.63±0.02	8.63±0.07	2468.00±12.34
21.3±0.1	21.0±0.1	50.6±0.1	42.8±0.1	41.0±0.1	31.2±0.1	4.17±0.02	6.03±0.02	10.02±0.07	2679.00±13.40
21.7±0.1	21.8±0.1	55.1±0.1	48.2±0.1	45.8±0.1	32.8±0.1	4.23±0.02	6.25±0.02	12.25±0.07	2851.00±14.26
27.9±0.1	23.1±0.1	58.7±0.1	51.4±0.1	49.4±0.1	35.6±0.1	4.20±0.02	6.65±0.02	11.96±0.07	3034.00±15.17
27.1±0.1	23.7±0.1	59.8±0.1	52.5±0.1	50.5±0.1	36.6±0.1	4.26±0.02	6.79±0.02	12.26±0.07	3076.00±15.38
26.9±0.1	13.5±0.1	41.6±0.1	33.0±0.1	30.5±0.1	23.8±0.1	2.12±0.02	4.61±0.02	7.64±0.07	2248.00±11.24
26.5±0.1	16.0±0.1	47.7±0.1	40.3±0.1	37.3±0.1	27.5±0.1	2.41±0.02	5.08±0.02	9.02±0.07	2529.00±12.65
34.0±0.1	13.7±0.1	54.2±0.1	49.4±0.1	45.6±0.1	36.4±0.1	2.11±0.02	5.15±0.02	10.82±0.07	2701.00±13.51
31.7±0.1	14.8±0.1	58.1±0.1	53.6±0.1	49.8±0.1	42.8±0.1	2.21±0.02	5.42±0.02	12.08±0.07	2850.00±14.25
28.6±0.1	9.9±0.1	49.3±0.1	44.2±0.1	39.9±0.1	34.4±0.1	2.74±0.02	4.65±0.02	9.53±0.07	2470.00±12.35
29.4±0.1	9.8±0.1	49.3±0.1	44.2±0.1	40.0±0.1	34.5±0.1	2.70±0.02	4.64±0.02	9.54±0.07	2469.00±12.35
31.3±0.1	11.5±0.1	54.1±0.1	49.6±0.1	45.7±0.1	39.0±0.1	2.90±0.02	4.89±0.02	10.88±0.07	2669.00±13.35

Also, the main processed properties such as the suction and vapour injection mass flows, are identified from the previous analysis using the provided data of Table 6. The performance indicators of the heat pump are calculated from the same dataset by means of Equation. (6)– Equation. (7) with the results presented in Table 7.

**Table 7.** Processed data of the heat pump.

Compressor suction mass flow rate (kg/s)	Vapour injection mass flow rate (kg/s)	Isentropic efficiency (-)	Volumetric efficiency (-)
0.0651±0.0023	0.0060±0.0014	0.36±0.01	0.91±0.03
0.0609±0.0025	0.0082±0.8150	0.42±0.16	0.86±0.03
0.0613±0.0026	0.0093±0.0016	0.45±0.01	0.83±0.03
0.0593±0.0027	0.011±0.0017	0.46±0.01	0.79±0.03
0.0547±0.0027	0.0138±0.0017	0.43±0.01	0.74±0.04
0.0552±0.0028	0.0141±0.0018	0.44±0.01	0.74±0.04
0.0417±0.0024	0.0093±0.0015	0.38±0.01	0.75±0.04
0.0424±0.0025	0.0119±0.0016	0.39±0.01	0.70±0.04
0.0237±0.0028	0.0147±0.0017	0.31±0.02	0.43±0.05
0.0206±0.0029	0.0166±0.0018	0.29±0.02	0.36±0.05
0.0198±0.0026	0.0135±0.0016	0.29±0.02	0.41±0.05
0.0199±0.0026	0.0135±0.0016	0.29±0.02	0.41±0.05
0.0178±0.0028	0.0158±0.0017	0.28±0.02	0.34±0.05

### Uncertainty analysis and final data set

In order to adequately analyse the obtained data set, an uncertainty analysis is provided using EES software ^31^. According to Reference 47, 48, in order to calculate the propagation of sensors accuracy through the calculated properties, the systematic error (ε) of a given calculated value (y) is identified by Equation. (17).


$$\;\varepsilon_{y}\text{=}\sqrt{\sum_{i}{\left(\frac{\partial y}{\partial x_{i}}\right)}^{2}\varepsilon_{x_{i}}^{2}}\;(17)$$

where ε _χi_ is the measurement uncertainty of the independent variables and $\frac{\partial y}{\partial x_{i}}$ the partial derivative of the function y with respect to every independent variable χ _i_.

Using Equation. (17) and taking into account the accuracies of Table 5, the uncertainties of the processed data are then calculated and are shown in Table 6 and Table 7.

## Results and discussion

The processed test results of the heat pump are initially presented over a sufficient range of temperatures. After that, the results of the verified semi-empirical model are given, identifying the compressor losses and their magnitude.

### Experimental results with R1234ze(E)

In this section, the experimental results of the heat pump configuration with R1234ze(E) are presented for variable condenser and evaporator temperatures. The test data ( Table 6) are used to determine the capacities of the components, for evaluating the overall system performance and compared with the operation with R407C based on the manufacturer data.

**Figure 8.**
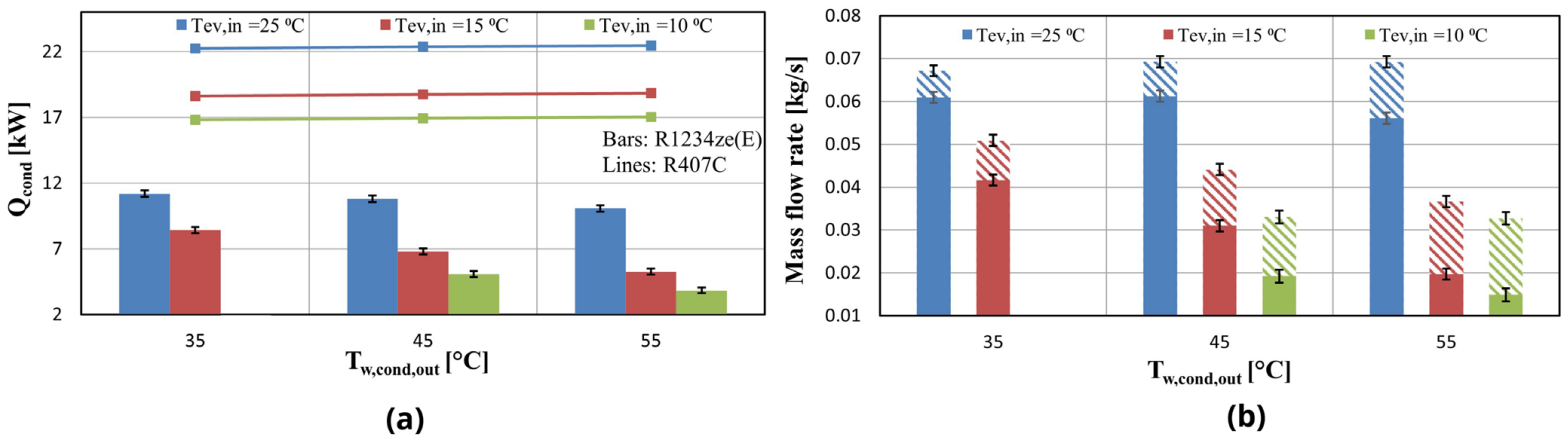
Condenser heat and compressor mass flow rates for the two refrigerants at three water supply temperature levels for variable condenser water temperature: ( **a**) Condenser heat; ( **b**) Compressor mass flow rate at the evaporator (solid bars) and vapor injection mass flow rate (dashed bars).

The delivered condenser heat for variable temperatures is depicted in Figure 8. The maximum heating capacity is 11 kW (50% lower compared to R407C) for evaporator inlet water temperature of 25 °C, while the minimum is observed for high temperature lifts between supply and sink temperatures (ΔT>30 K) providing a heating capacity of 4 kW at the maximum lift of 45 K. On the other hand, the capacity seems not to be affected with R407C for the same evaporation temperature level, with the increase becoming more significant as the temperature lift increases.

The heating capacity is also highly affected by the supply temperature level, which drastically varies the mass flow rate. The suction mass flow rate is reduced by over 8% when the temperature lift increases from 20 to 30 K for an evaporator temperature of 25 °C while the same reduction for an evaporator temperature of 15 °C is over 25%. On the other hand, the vapour injection mass flow rate is increased by at least 23% for these temperature lifts. Despite the contribution of the vapour injection, the total mass flow rate is lower than the expected. According to manufacturer performance data provided for R407C there is an observed mass flow decrease of over 50% for similar operating conditions with the experimental ones.

**Figure 9.**
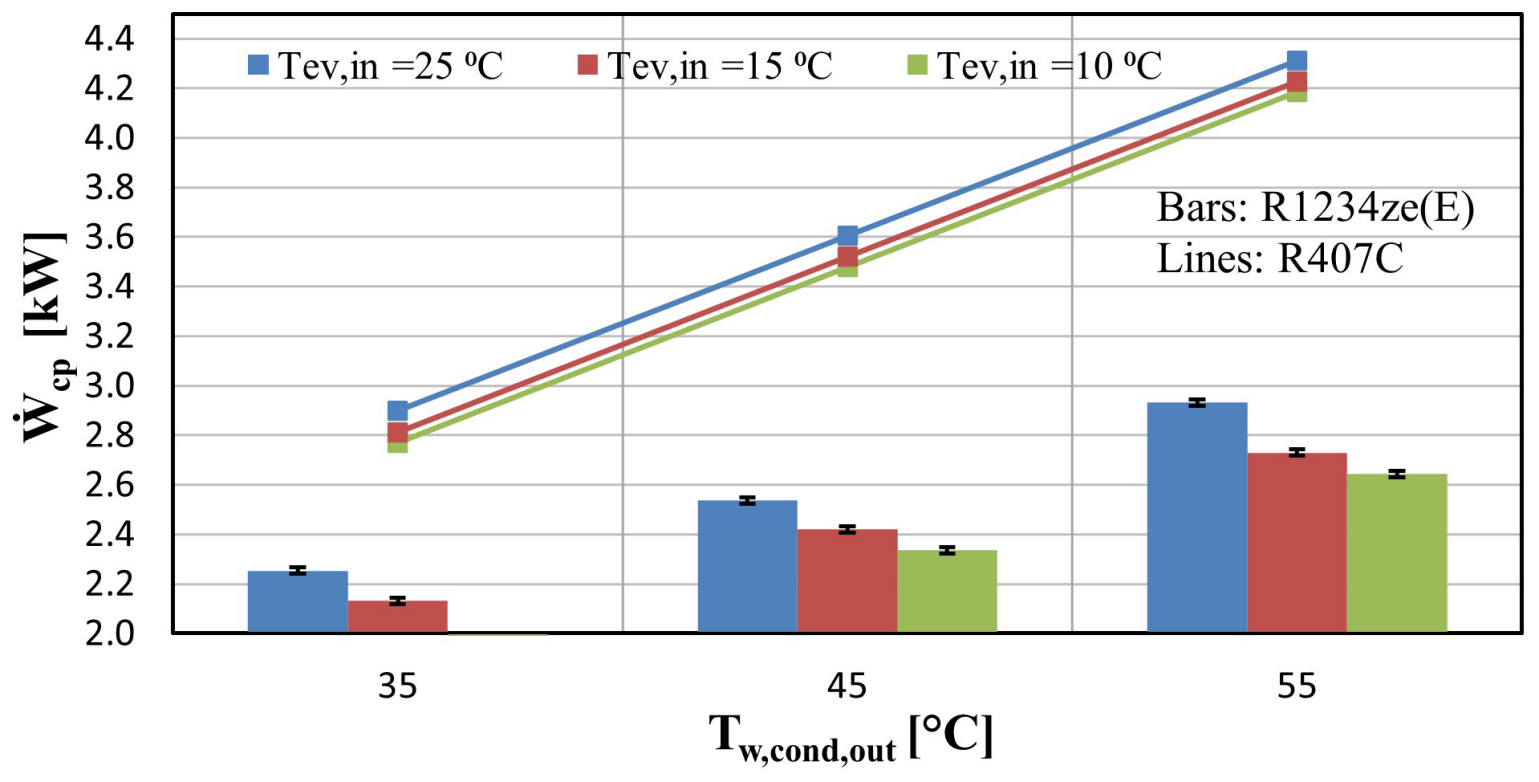
Compressor power (Ẇ _cp_) for the two refrigerants at three water supply temperature levels and for variable water condenser temperature.

The electricity consumption of the compressor of the heat pump is depicted in Figure 9, and indicates that it increases in an almost linear way when the pressure ratio is elevated (higher temperature lifts). This is a reasonable trend, since for higher pressure ratios the power requirements are increased in order to achieve the desirable discharge pressure. This leads to an increase of the vapour injection mass flow rate, accordingly reducing the suction flow rate. Also, from Figure 9 is clear that the compressor underperforms with R1234ze(E) since for the same operating conditions, the power consumption with R407C is 0.6-1 kW greater. This follows the significantly lower capacity observed in Figure 8, due to the lower refrigerant mass flow rates.

**Figure 10.**
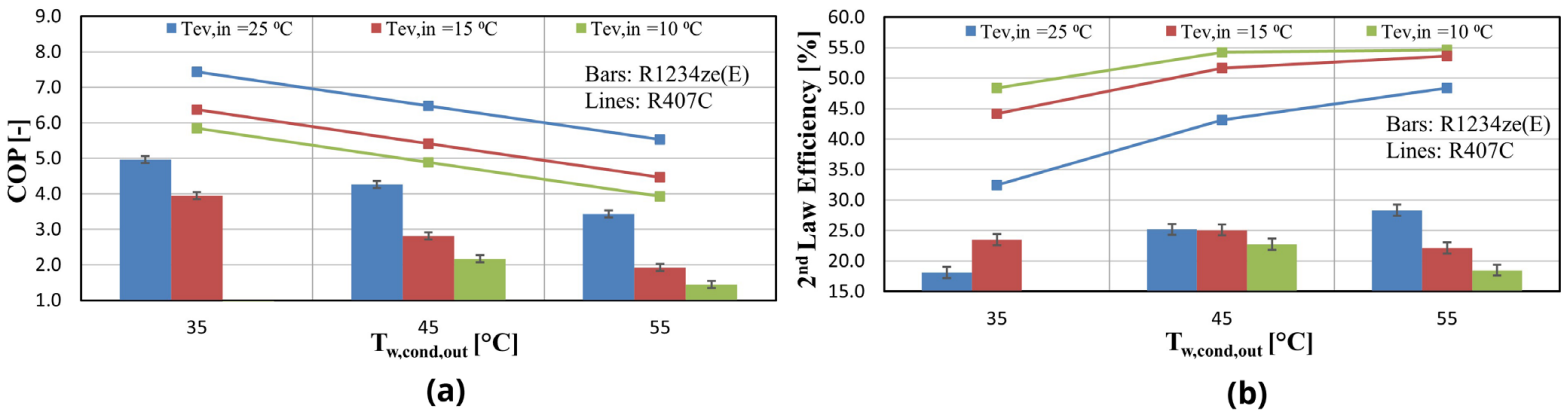
Compressor coefficient of performance (COP) and 2 ^nd^ law efficiency for the two refrigerants at three water supply temperature levels for variable water condenser temperature: ( **a**) COP; ( **b**) 2 ^nd^ law efficiency.

The heat pump performance is expressed through the COP, considering the compressor power only. The power of all auxiliaries (water pumps, inverter losses, etc.) has also been measured and is equal to about 140 W. The compressor COP together with the 2 ^nd^ law efficiency are presented in Figure 10 for the same operating conditions as before ( Figure 9).

The heat pump reaches a maximum COP of 5 for a low lift of 10 K, whereas for a lift of 20 K the COP is about 4 for all condenser temperatures. For the same temperature lifts, the COP values based on the manufacturer data are 7.1 and 5.5 respectively. For high-temperature lifts of over 40 K the COP is decreased reaching values below 2, which is much lower than expected (about 4 with R407C). The 2 ^nd^ law efficiency assists in evaluating the low performance of the compressor. This efficiency is in the range of 18.1–28.33%, while it is increased to about 40–50% with the same compressor when using R407C, which is a typical value ^49^.

According to the initial design work of the system concerning the compressor selection procedure, the COP was expected to be decreased by 5–10% compared with R407C, but the test results revealed a much lower performance, with the COP being 50% lower than expected. The reasons behind this are going to be identified and explained later, using the developed semi-empirical model.

### Results of the developed semi-empirical model with vapour injection

The developed model is initially calibrated and its results compared with the available data from the manufacturer with R407C and the same compressor (ZH13KVE) for verification purposes. As a second step, the model is fine-tuned based on the test data, identifying the main reasons for underperformance.

***Vapour injection semi-empirical model verification with R407C.*** The operating conditions consider a suction superheat of 10 K, condenser subcooling of 3 K, and frequency of 50 Hz. By doing so, 36 operating datasets ^50^ are extracted, covering compressor suction temperatures from -15 to 25 °C and condensing temperatures from 25 to 60 °C, all within the compressor envelope.

The semi-empirical model parameters are then fine-tuned ^37^, when using the standard suction pressure drop model (with a constant friction factor) and the improved model (with the logarithmic correlation presented previously).

The calibration work resulted in the same values of the parameters with the standard and improved suction pressure drop models, with the exception of the two friction factor parameters. The values of all parameters are shown in Table 8.

**Table 8.** Semi-empirical model parameters with vapour injection and R407C.

Parameter	Sub-processes	Standard suction pressure drop	Improved suction pressure drop	Units
*AU _amb,cp_*	Heat transfer coefficient for heating-up during supply	12.6		W/K
*AU _ex,cp,n_*	Heat transfer coefficient for heat loss during the exhaust	11.72		W/K
*AU _su,cp,n_*	Heat transfer coefficient for heat losses to the ambient	218.7		W/K
*A _leak, cp_*	Effective area of leakages	6.41E-08		m ^2^
*Ẇ _loss0,cp_*	Constant term of the electrical power losses	276.1		W
*α _cp_*	Coefficient of the variable term of the electrical power losses	0.227		-
*V _s,cp_*	Swept volume of the compressor	71.03		cm ^3^
*r _v,in_*	Built-in volume ratio of the compression	3.54		-
*K*	Lumped friction factor parameter	3.99E+08	-	1/m ^4^
$A_{0}^{\prime }$	Constant term of the friction factor correlation adjusted parameter	-	3.401E+08	1/m ^4^
$A_{1}^{\prime }$	Variable term of the friction factor correlation adjusted parameter	-	5.114E+06	1/m ^4^

Significant heating of the refrigerant at the suction is observed, since the suction heat transfer coefficient is one order of magnitude higher than the other two heat transfer coefficients. In addition, there is an over-estimation of the swept volume of the compressor by 5.6%, which is also observed in the findings of relevant literature ^27, 30, 33^. This is because the identified swept volume does not correspond to the actual one, but considers several other processes. The built-in volume ratio r _v,in_ *,* is also an identified parameter of the model and represents the ideal volume ratio of the compressor.

Due to the compatibility of R407C and the examined compressor with all operating conditions included in the envelope, the suction fluid flow is a fully developed turbulent flow. This is identified by the parameter values of the logarithmic expression for suction pressure drop that gives an almost constant friction factor, and thus expecting the results of the two models to be similar. Specifically, the $A_{1}^{\prime }$ parameter is related to the logarithmic part of Equation. (13) and is two orders of magnitude lower than the $A_{0}^{\prime }$ , which represents the constant part of the correlation with a value similar to the constant friction factor (K).

**Figure 11.**
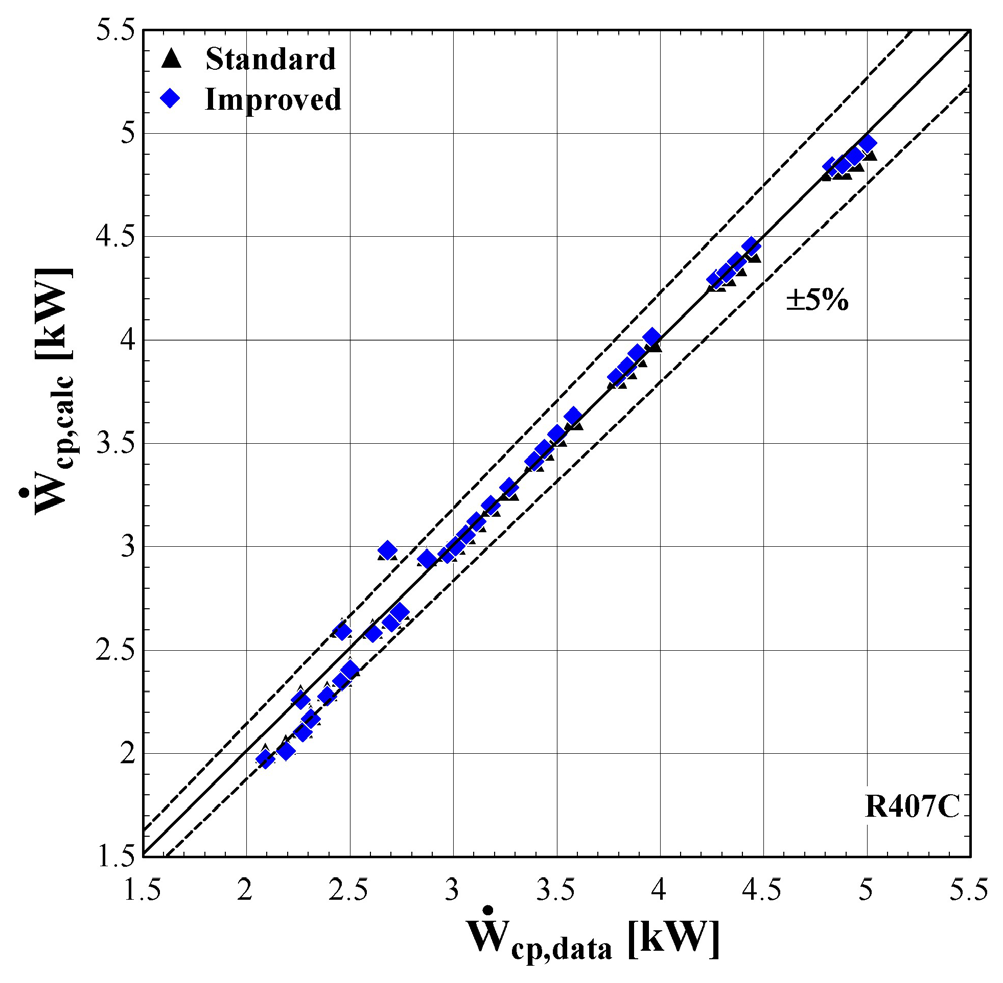
Comparison of the calculated electrical power, Ẇ _cp,calc_ (with the standard and improved suction pressure drop) with the available one (Ẇ _cp,data_).

The results of the semi-empirical model using the standard and improved models for suction pressure drop are depicted in Figure 11– Figure 13 comparing the calculated mass flow rate, the electric power and the discharge temperature of R407C with all available data from the manufacturer. The 5% margins for electric power and mass flow rate and 3 K margin for the discharge temperature are also indicated.

**Figure 12.**
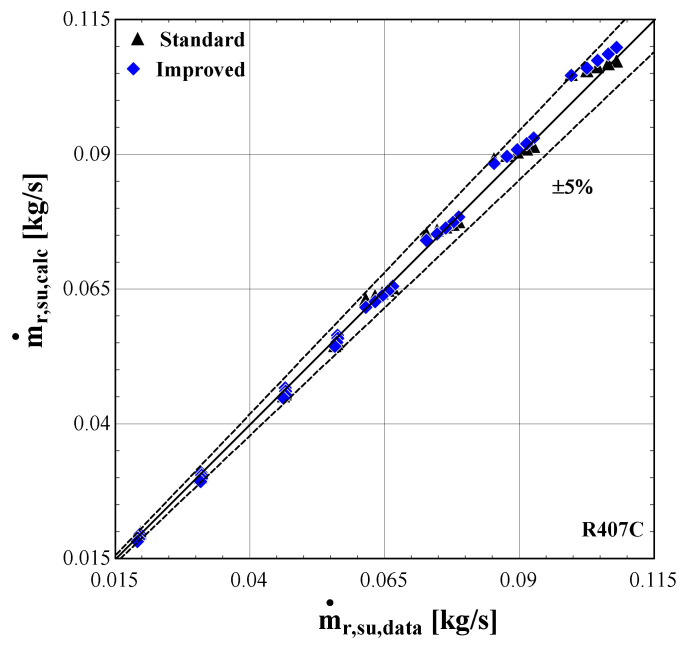
Comparison of the calculated mass flow rate, *ṁ*_r,su,calc_ (with the standard and improved suction pressure drop) with the available one ( *ṁ*_r,su,data_).

**Figure 13.**
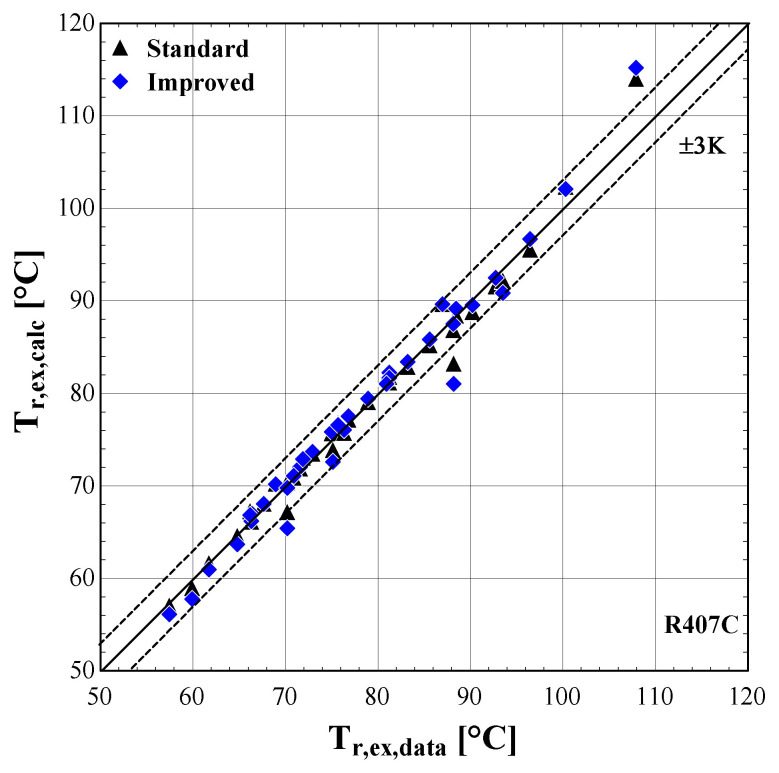
Comparison of the calculated discharge temperature, T _r,ex,calc_ (with the standard and improved suction pressure drop) with the available one (T _r,ex,data_).

As shown in Figure 11, the electric power prediction for the compressor is sufficiently calculated for the whole range, keeping the deviation within ±5% for the majority of testing points, observing very few diversions. These are to be expected, since the calibration process has been accomplished for a very large range of conditions. However, the electric power prediction for moderate and high values is almost identical to the available one. This is achieved for both standard and improved suction pressure drop models, since the mass flow rate and refrigerant velocity is increased, assuring the fully developed flow of the refrigerant.

As far as the mass flow rate is concerned, the prediction is always within a 5% margin showing better accuracy for low and moderate rates (below 0.07 kg/s). Comparing the results of the standard and improved suction pressure drop models, it is evident that there is no clear improvement since the values are almost identical.

Finally, the discharge temperature is adequately calculated with most of the operating conditions within the 3 K range. Only two conditions of moderate discharge temperature and one of high temperature exceed the ±3 K limit, being underestimated by almost 6 K. This discrepancy can be explained because these points are characterized by very low evaporating temperatures (below -10 °C) and are located in the boundaries of the compressor envelope making it very challenging for the model to accurately predict the behaviour. The results of the two suction pressure drop models are similar, with the improved model having a better accuracy for moderate temperatures in the range of 70–90 °C.

The minimum, maximum and average deviation of the predicted variables for both standard and improved suction pressure drop model are presented in Table 9.

**Table 9.** Minimum, maximum, and average deviation of the predicted variables of the semi-empirical model compared with the available data for R407C.

	Discharge temperature deviation (K)			Mass flow rate deviation (%)			Electric power deviation (%)		
	Min	Max	Average	Min	Max	Average	Min	Max	Average
**Standard model**	-5.88	5.06	1.07	-4.47	3.43	1.77	-11.30	6.96	1.99
**Improved model**	-5.41	5.43	1.06	-5.52	2.61	1.54	-11.23	6.85	1.97

Although there are a few values that have greater deviations than the defined limits, in all cases the average error is kept below 2% for the mass flow rate and electric power respectively, and 1.07 K for the discharge temperature. The accuracy of the two suction pressure drop models seems to be the same with negligible differences, as indicated by the average deviations of Table 9.

Finally, the verification of the developed model is proved by the low deviation values for the main properties. These deviations could be even lower, if the range of the conditions were restricted, for example by considering compressor suction temperatures from -10 to 15 °C and condensing temperatures from 40 to 60 °C. Moreover, the results of the two suction pressure drop models are almost identical, indicating that the use of the improved model does not bring any improvements at these specific conditions.

***Semi-empirical model calibration to experimental data with R1234ze(E) and the effect of suction pressure drop.*** The vapour injection semi-empirical model is then calibrated with the use of the test data. The inputs concern the refrigerant suction pressure and temperature, the discharge pressure and the vapour injection pressure, temperature and mass flow rate ( Table 6 and Table 7). This calibration process is followed for both the standard and improved suction pressure drop approach. The fine-tuned values of all parameters are given in Table 10, which significantly differ from the ones for R407C presented in Table 8.

**Table 10.** Semi-empirical model parameters with vapour injection and R1234ze(E).

Parameter	Sub-processes	Standard suction pressure drop	Improved suction pressure drop	Units
*AU _amb,cp_*	Heat transfer coefficient for heating-up during supply	1600		W/K
*AU _ex,cp,n_*	Heat transfer coefficient for heat loss during the exhaust	320.8		W/K
*AU _su,cp,n_*	Heat transfer coefficient for heat losses to the ambient	10.02		W/K
*A _leak, cp_*	Effective area of leakages	1.77E-07		m ^2^
*Ẇ _loss0,cp_*	Constant term of the electrical power losses	487.8		W
*α _cp_*	Coefficient of the variable term of the electrical power losses	0.5496		-
*V _s,cp_*	Swept volume of the compressor	63.43		cm ^3^
*r _v,in_*	Built-in volume ratio of the compression	4.726		-
*K*	Lumped friction factor parameter	8.35E+08	-	1/m ^4^
$A_{0}^{\prime }$	Constant term of the friction factor correlation adjusted parameter	-	-2.3975E+10	1/m ^4^
$A_{1}^{\prime }$	Variable term of the friction factor correlation adjusted parameter	-	1.2593E+12	1/m ^4^

The suction’s overall heat transfer coefficient is rather low, leading to a minor heating up of the refrigerant by about 1–2 K. This is because the mass flow rate of the vapour injection is increased, reducing at the same time the suction flow rate. Also, overall heat transfer coefficient of the discharge is high, which enhances the heat losses of the compressed refrigerant, although the ambient temperature was even 30 °C in some cases.

In addition, the electric compressor losses are also higher with R1234ze(E) than with R407C, presenting a 76% increment on the constant electrical losses factor (Ẇ _loss0,cp_) and an increase of over 100% for the variable losses factor (α _cp_). This was expected to some extent, since the constant inverter losses of the tested compressor with R1234ze(E) are included in the measured electric power. Moreover, the energy balance at the compressor’s shell imposes a very high heat transfer coefficient at the casing. This could indicate that the assumption of the isothermal wall with a constant temperature is not entirely valid, and possibly the shell should be split into two regions (top and bottom), each with a different envelope temperature.

The friction factor of the standard suction pressure drop approach has a similar value to the case with R407C (although two times higher). On the other hand, the improved model gives a parameter of the logarithmic part two orders of magnitude greater than the constant part, revealing a significant variation in the friction factor as the evaporation temperature changes. This will become clearer later in this work, when the compressor losses are examined.

**Figure 14.**
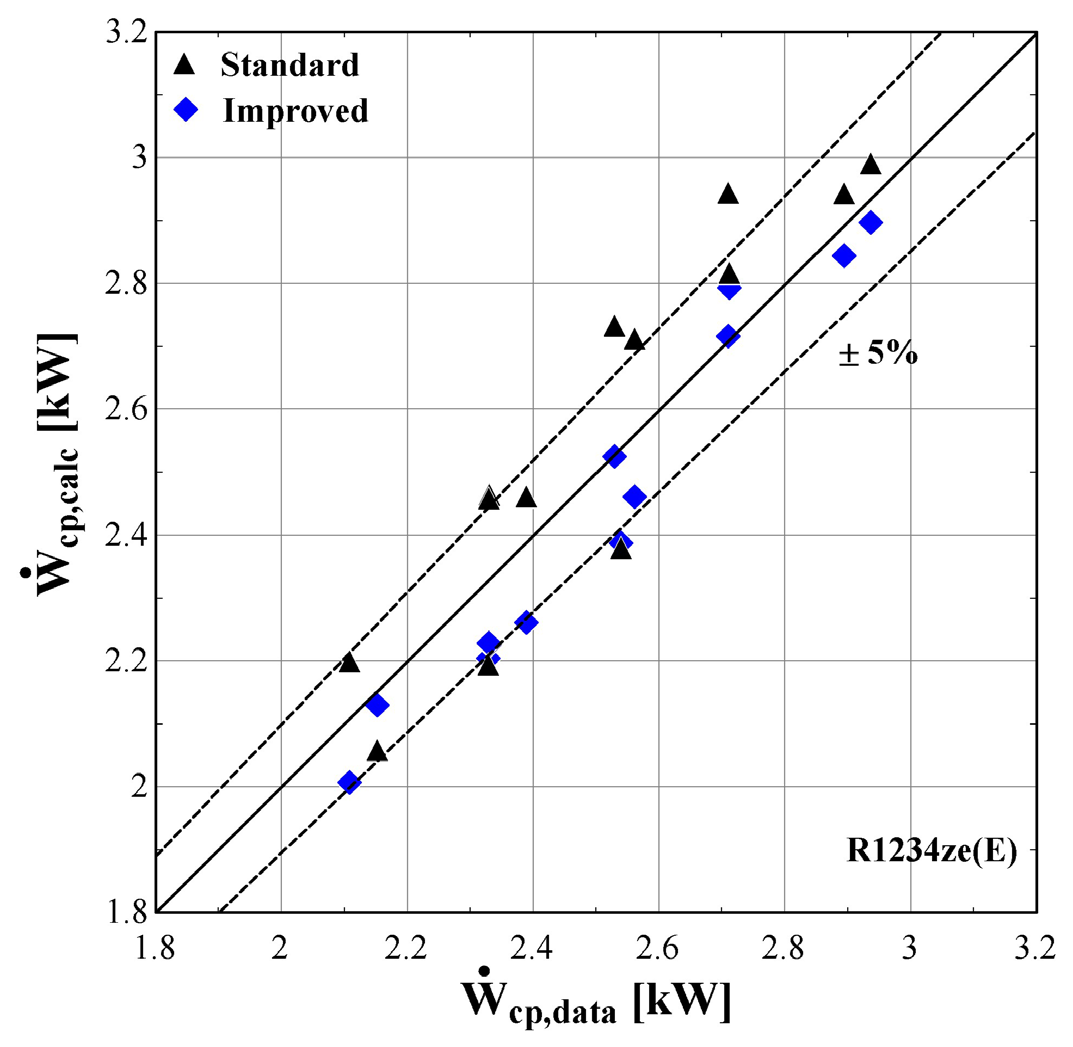
Comparison of the calculated electrical power, Ẇ _cp,calc_ (with the standard and improved suction pressure drop) with the measured one (Ẇ _cp,data_).

Figure 14 – Figure 16 compare the model results of the standard and improved friction factor approach with the measured data.

**Figure 15.**
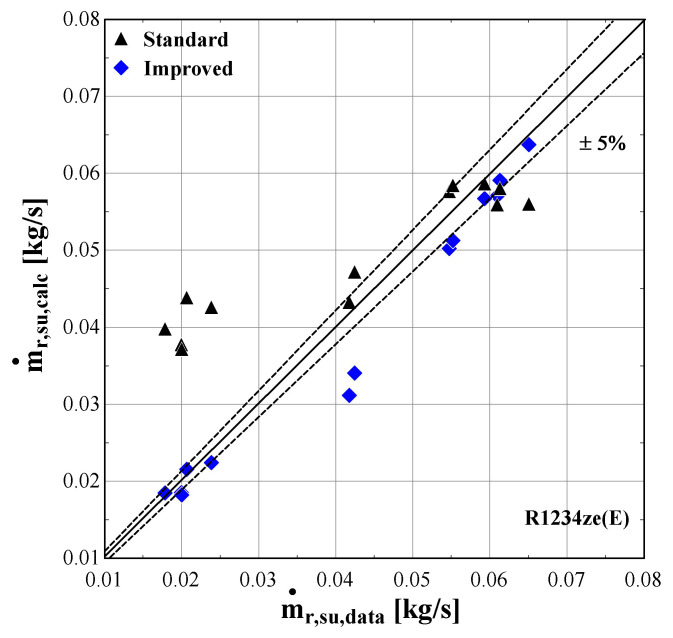
Comparison of the calculated mass flow rate, ṁ _r,su,calc_ (with the standard and improved suction pressure drop) with the measured one (ṁ _r,su,data_).

**Figure 16.**
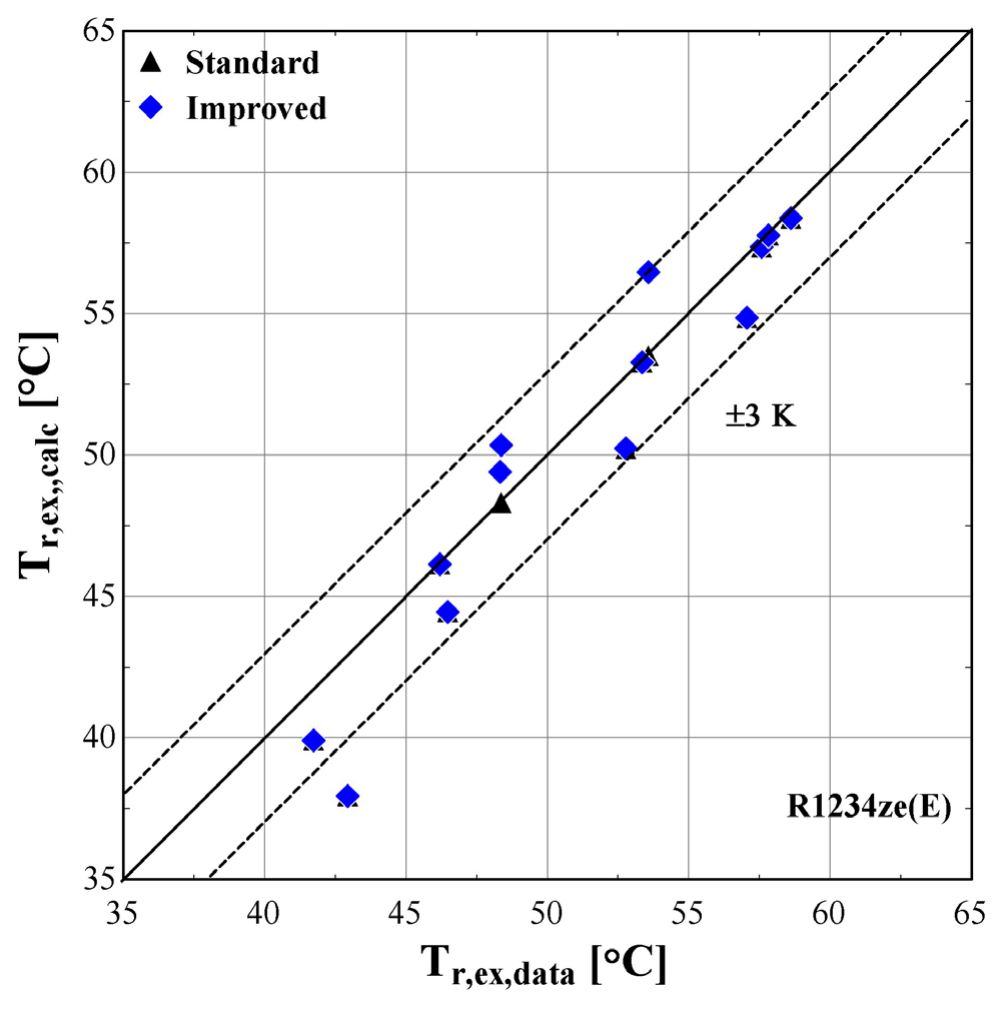
Comparison of the calculated discharge temperature, T _r,ex,calc_ (with the standard and improved suction pressure drop) with the measured one (T _r,ex,data_).

The electric power calculated by the improved model ( Figure 13) seems to be consistent with the experimental results always staying within the ±5%. Moreover, the majority of examined conditions are closer to the experimental data than the data obtained using the standard model.

The standard suction pressure drop introduces large deviations to the mass flow rate, especially for the lower values, whereas for moderate/high flow rates (in the range of 0.04-0.06 kg/s) the results are similar to the measured ones. On the other hand, the improved suction pressure drop model reduces the discrepancies in the whole range. Especially for the lower mass flow rates, the error is significantly reduced, with the calculations ranging within the ±5% bandwidth. For the moderate mass flow rates, the logarithmic trend of the suction pressure drop seems to be less accurate, showing a maximum underestimation of about 25%. This can be explained from the calibration process that aims to minimize the total error, with the algorithm compromising between reaching a lower accuracy for these conditions and a higher accuracy for conditions within low and high mass flow regions.

The calculated discharge temperature with the two suction pressure drop models show similar results, with a small deviation from the measured values. The majority of examined conditions are within the ±3 K range, except for one that almost reaches a 5 K underestimation and corresponds to an extreme condition with a very low pressure ratio and temperature lift (discharge temperature of 43 °C).

**Figure 17.**
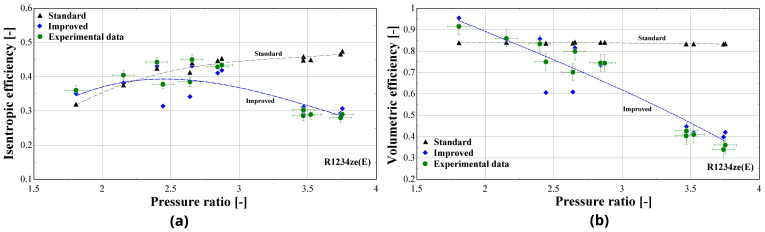
Comparison of the calculated efficiencies (with the standard and improved suction pressure drop) with the available ones from the test data: ( **a**) Isentropic efficiency; ( **b**) Volumetric efficiency.

In order to further examine the impact of the improved model on the overall prediction accuracy, the isentropic and volumetric efficiencies are calculated and presented in Figure 17, the trendline of each dataset is also shown (3 ^rd^ order polynomial for the isentropic efficiency and 2 ^nd^ order polynomial for the volumetric efficiency, also well approached with a linear correlation).

Both standard and improved friction factor models adequately predict the isentropic efficiency in a pressure ratio range from 1.5 to 3. This range corresponds to higher mass flow rates with a low suction pressure drop. For higher pressure ratios, the accuracy of the improved model improves, with its results closely following the experimental data that reach an isentropic efficiency of just 30%.

Regarding the volumetric efficiency, the standard model only provides adequate accuracy for low pressure ratios (below 2.5), while its calculations lead to a constant volumetric efficiency for all conditions, equal to about 85%, instead of declining with the pressure ratio, with the maximum deviation exceeding 140%. The results of the improved model are very close to the experimental values for the whole operating range, capturing the linear reduction of the volumetric efficiency with the pressure ratio. Finally, the very low volumetric efficiency of even 40% is the result of the high suction pressure drop, being the main cause of the compressor underperformance, as will be discussed later.

The previous outcomes are supported by the minimum, maximum and average deviations of the three key properties considering the two suction pressure drop correlations, given in Table 11.

**Table 11.** Minimum, maximum and average deviation of the predicted variables of the semi-empirical model with standard and improved suction pressure drop approach compared to the measured data with R1234ze(E).

	Discharge temperature deviation (K)			Mass flow rate deviation (%)			Electric power deviation (%)		
	Min	Max	Average	Min	Max	Average	Min	Max	Average
**Standard**	0.05	4.98	1.10	-123.0	14.00	41.80	-8.63	6.29	5.01
**Improved**	-2.90	4.98	1.54	-4.46	25.34	8.20	-3.02	5.95	3.20

The deviation of the discharge temperature is about the same for both cases, and similarly for the electric power. However, the mass flow rate deviation is significantly decreased when using the improved suction pressure drop approach, as the average deviation is reduced by 75%, compared to the standard model. This indicates that the new model approach for suction pressure drop recreates the experimental points more accurately, since for pressure ratios above 2.5 the flow is closer to a laminar one with high friction factor rather than the standard turbulent conditions. Therefore, the improved model will be used from now on, so that to evaluate the compressor losses with a higher accuracy.

**Figure 18.**
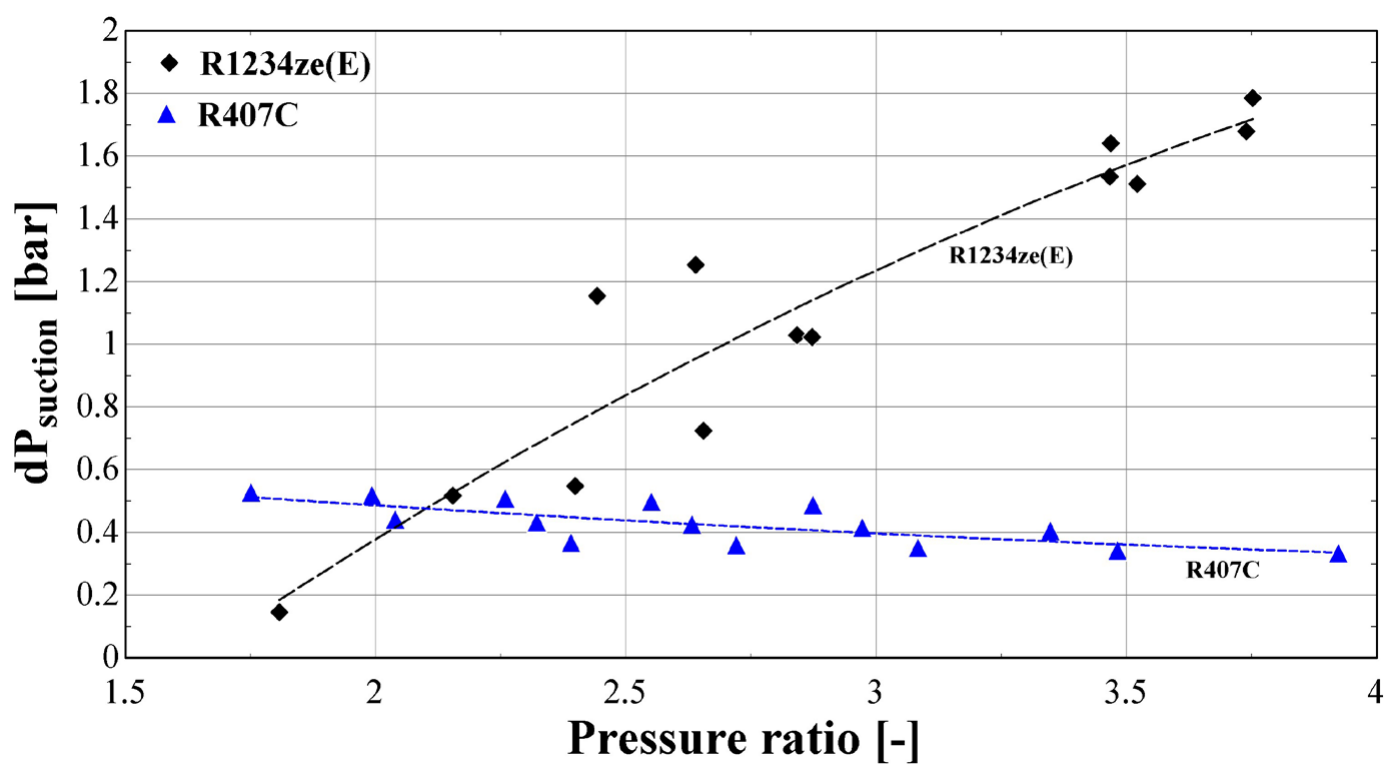
Calculated suction pressure drop (dP _suction_) of R407C and R1234ze(E) as a function of the pressure ratio.

***Compressor losses.*** The previous analysis leads to identifying the losses mechanisms, which are related to the pressure drop, heat losses and electrical losses. Figure 18 presents the suction pressure drop of the same compressor with R407C and R1234ze(E), when using the improved model. In order to perform an impartial comparison between the two refrigerants, a subset of the initial data set of R407C is obtained, which corresponds to the same range of evaporating and condensing temperatures with the test data of R1234ze(E).

The suction pressure drop with R1234ze(E) ranges from about 0.2 bar for low temperature lifts, reaching 1.8 bar for a pressure ratio of 3.8. This drop is very high and has a significant impact on the refrigerant density, leading to a very low volumetric efficiency, as presented in Figure 17. On the other hand, the pressure drop of R407C varies within a reasonably small range (0.3-0.6 bar), and slightly decreases for larger pressure ratios. It should be highlighted that the discharge pressure drop of the two refrigerants is similar and always lower than 0.2 bar. This drop is within the reported values found in the literature ^29, 49^, and has a negligible impact on the compressor performance.

**Figure 19.**
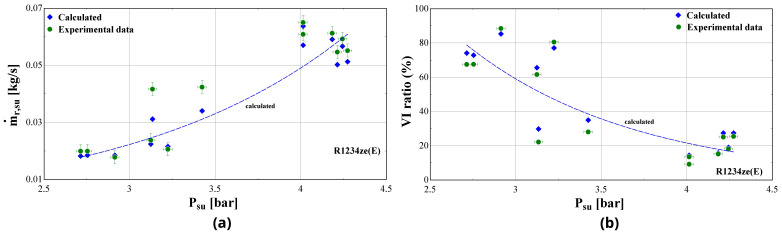
Suction mass flow rate (ṁ _r,su_) and vapour injection mass flow rate ratio (VI ratio) as a function of the suction pressure (P _su_) of R1234ze(E): ( **a**) Suction mass flow rate; ( **b**) Vapour injection mass flow rate ratio.

The above results reveal the main reason for compressor underperformance which is related to the low suction pressure of R1234ze(E) for low evaporation temperatures. The suction pressure is about 3–4 bar less than with R407C for the same temperature levels (although the pressure ratio range is very similar), indicating that the flow approaches the laminar regime due to a reduced fluid velocity and Reynolds number, increasing, in that case, the friction factor as presented previously. The result is a very high pressure drop at the suction line of the compressor, resembling the operation of a choking valve that imposes the refrigerant to flow through the alternative path of the vapor injection line. This is depicted in Figure 19 and shows the measured and calculated suction mass flow rate and the mass flow rate ratio (vapour injection-VI to suction flow) as a function of the suction pressure for R1234ze(E) refrigerant.

The ratio between injected and suction mass flow rate of R1234ze(E) is extremely high reaching about 80% at low suction pressure and temperature, whereas it reduces to typical values of about 20–30% for a lower pressure ratio. This is still rather high compared with the one of R407C that ranges from about 7 to 36%.

**Figure 20.**
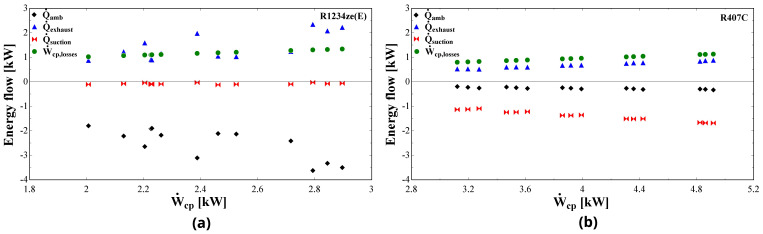
Energy flows (heat losses to the ambient: $\dot{\text{Q}}$ _amb_, heat losses to the exhaust: $\dot{\text{Q}}$ _exhaust_, heat gain at the suction: $\dot{\text{Q}}$ _suction_, compressor losses: Ẇ _cp,losses_) at the fictitious isothermal wall as a function of the compressor power (Ẇ _cp_): ( **a**) R1234ze(E); ( **b**) R407C.

Apart from the pressure losses, the energy flows of the compressor are examined. These are related to the energy balance at the fictitious isothermal wall (negative values once leaving the shell, positive ones to the shell), involving the suction heat gain, discharge heat loss, ambient heat losses and electrical losses. These are presented in Figure 20 for both R1234ze(E) and R407C.

The thermal and electrical losses with R1234ze(E) are significantly higher compared with the ones of R407C, which is a direct outcome of the higher parameter values identified during the model calibration ( Table 7 and Table 9). Almost all variables are linearly related to the compressor work with R407C, while both thermal and electrical losses are in close agreement with the results presented in Reference 32.

The heat transfer at the suction in the case of R1234ze(E) has a minor effect on the overall energy balance with negligible heating of the refrigerant. This is not the case with R407C, for which the 35% of the compressor power heats the supplied refrigerant. Moreover, the electrical losses have a linear dependency on the compressor work and their values are about the same for both refrigerants and range between 0.8 and 1.3 kW. However, the relative electrical losses are much lower with R407C, due to its higher compressor power.

The heat transfer at the exhaust as well as the ambient losses with R1234ze(E) dominate the energy balance applied to the compressor casing. The cooling down of the refrigerant takes values from 0.9 to 2.3 kW which corresponds to 45–76% of the total compressor work, while this fraction is reduced for R407C to 10–20%. This reveals that due to the significant pressure drop of R1234ze(E) the temperature is reduced and only a small fraction of the total supplied energy is transferred to the refrigerant. In addition, the heat of the R1234ze(E) cooling down and the electrical losses are practically transferred to the surrounding environment, since their sum is about the same with the ambient losses which are in the range of 1.9-3.6 kW. These losses are much lower for R407C, due to the minor refrigerant cooling down and the increased suction heating up, as it was mentioned before.

## Conclusions

An experimental setup of a vapour injection scroll compressor of a heat pump has been presented. This water-to-water heat pump is for heating only and designed for R1234ze(E) refrigerant. The testing procedure focused on a wide variety of operating conditions with the water supply temperatures ranging from 10 °C to 25 °C and sink temperatures from 32 °C to 55 °C. The test results were evaluated, revealing that the compressor was not performing as expected, especially for high temperature lifts (ΔT>30 K). At such conditions, the heating capacity was drastically reduced accompanied by a significant decrease in the COP, which reached values below 2. This underperformance was also identified by the values of the 2 ^nd^ law efficiency that reached a maximum of just 28.33%.

In order to identify the main causes of this and examine with detail the losses mechanisms, a semi-empirical model for scroll compressors with vapour injection has been developed by the authors, including an improved version of the suction pressure drop approach that considers both the laminar and turbulent flow regimes. The accuracy of this model has been verified based on the same compressor with R407C, with the standard and improved model for the suction pressure drop giving similar results. This is because the suction mass flow rate is high enough so that the inlet fluid flow remains turbulent over the whole range of suction temperatures. The next step included the semi-empirical model calibration based on the experimental tests with R1234ze(E). Comparing the predicted values of the model with the experimental ones, it was shown that the discharge temperatures are sufficiently estimated with a deviation lower than ±5 K for both the standard and improved model, with a similar accuracy for the electric power. However, the greatest improvement, in the improved model, is observed to the suction mass flow rate, since the error is reduced by almost 80% compared with the constant friction factor approach. The analysis shows that the properties of R1234ze(E) related to a lower suction pressure and density reduce the flow velocity and Reynolds number, with the flow approaching (or reaching) the laminar regime, increasing the friction factor and triggering the large pressure losses at the suction line, which become up to five times higher than the ones of R407C.

Furthermore, the energy balance at the fictitious isothermal wall of the compressor was examined for both refrigerants further enlightening the reasons for compressor underperformance with R1234ze(E). The thermal and electrical losses with R1234ze(E) are significantly higher compared with the losses of R407C including increased cooling down of the refrigerant at the discharge as well as very high ambient losses. Moreover, although the simulation with R407C led to 60% higher compressor power for similar operating conditions, the electrical losses were about the same with R1234ze(E), something that further decreased the overall compressor performance. As a result, only a small fraction of the total supplied energy is transferred to the refrigerant and the parameter values of the semi-empirical model divert from the typical ones of the literature, in order for the compressor to be modelled with the highest possible accuracy.

Overall, the proposed semi-empirical model for scroll compressor with vapour injection is verified with the improved correlation for suction pressure drop modelling, increasing both the physical background and prediction accuracy. This enables the prediction of the key properties of the compressor when operating at unexplored conditions even outside the envelope and with refrigerants that no performance data exist, with the final outcome to locate the losses related to the pressure drop, heat transfer and electrical consumption.

## Ethics and consent

Ethical approval and consent were not required.

## Nomenclature

**Table:** 

A	Area of heat exchanger	m ^2^
$A_{0}^{\prime }$	Constant parameter of the improved model	m ^-4^
$A_{1}^{\prime }$	Parameter of the logarithmic part of the improved model	m ^-4^
c _p_	Specific heat capacity	kJ·kg ^-1^K ^-1^
D	Diameter	m
f	Darcy-Weisbach friction factor	-
h	Enthalpy	kJ·kg ^-1^
K	Friction factor parameter	m ^-4^
ṁ	Mass flow rate	kg·s ^-1^
N	Compressor speed	s ^-1^
P	Pressure	bar
Q	Heat	kW
R	Ratio	-
Re	Reynolds number	-
T	Temperature	°C
U	Overall heat transfer coefficient	kW·m ^-2^K ^-1^
u	Velocity	m·s ^-1^
V	Volume	m ^3^
$\dot{\text{V}}$	Volumetric flow rate	m ^3^·s ^-1^
v	Specific volume	m3·kg ^-1^
w	Specific work	kJ·kg ^-1^
Ẇ	Electrical power	kW
X	Modified Reynolds number	m
Z	Number of data sets	-

**Table:** Greek symbols

α	Compressor losses variable term	-
Δ	Difference	-
ε	Relative roughness	m
η	Efficiency	-
Θ	Optimization parameter	-
μ	Dynamic viscosity	Pa·s
ρ	density	kg·m ^-3^

**Table:** Subscripts

0	Initial value
ad	Adapted conditions
amb	Ambient
c	Condenser
calc	Calculated value
cp	Compressor
data	Obtained value
ev	Evaporator
ex	Exhaust
in	Internal or inlet
inj	Injection
int	Intermediate
is	Isentropic
leak	Leakage
loss	Compressor losses
n	Nominal
out	Outlet
r	Refrigerant
s	Swept
su	Suction
v	Volumetric, isochoric
vi	Vapour injection
vol	Volumetric
w	Wall

**Table:** Abbreviations

COP	Coefficient of performance
EES	Engineering equation solver
GWP	Global warming potential
HC	Hydrocarbon
HEX	Heat exchanger
HFC	Hydrofluorocarbon
HFCO	Hydrochlorofluoroolefin
HFO	Hydrofluoroolefin
HTHP	High temperature heat pump
IHX	Internal heat exchanger
ODP	Ozone depletion potential
PLC	Programmable logic controller
PWM	Pulse Width Modulation
VI	Vapour injection

## Data Availability

Zenodo: Test and numerical data of a vapour-injection scroll compressor in a heat pump with R1234ze(E), [Dataset] https://doi.org/10.5281/zenodo.5785939 ^50^. This project contains the following underlying data: Test and simulation data_Compressor_with_VI_R1234ze(E).xlsx (all data used to produce the results of this study). Data are available under the terms of the Creative Commons Zero "No rights reserved" data waiver (CC0 1.0 Public domain dedication).
