# Supplementary material for: Identifying the performance and losses of a scroll compressor with vapour injection and R1234ze(E)
Source: Open Res Eur. 2022 Nov 28;2:49. Originally published 2022 Apr 20. [Version 2] doi: 10.12688/openreseurope.14658.2 (PMC10445871; doi:10.12688/openreseurope.14658.2)
Supplement: Peer review history

# Peer review: Reviewer response for version 2

**DOI:** 10.21956/openreseurope.16569.r30425

**Published:** 2022-12-07

**Authors:** Christoph Wieland

**Affiliations:** 1 Chair of Energy Systems, School, Technical University of Munich, Garching, Germany

My comments have been perfectly addressed. Thank you very much!

Is the study design appropriate and does the work have academic merit?

Yes

Is the work clearly and accurately presented and does it cite the current literature?

Yes

If applicable, is the statistical analysis and its interpretation appropriate?

Partly

Are all the source data underlying the results available to ensure full reproducibility?

Yes

Are the conclusions drawn adequately supported by the results?

Yes

Are sufficient details of methods and analysis provided to allow replication by others?

Yes

Reviewer Expertise:

Organic Rankine cycle power systems with expertise in both, experimental and numerical.

I confirm that I have read this submission and believe that I have an appropriate level of expertise to confirm that it is of an acceptable scientific standard.

# Peer review: Reviewer response for version 1

**DOI:** 10.21956/openreseurope.15834.r29493

**Published:** 2022-08-23

**Authors:** Christoph Wieland

**Affiliations:** 1 Chair of Energy Systems, School, Technical University of Munich, Garching, Germany

A scroll compressor designed for R407C has been investigated for R1234ze(E). The main feature of the comppressor is its capability of vapor injection in between the compression process. This increases the efficiency by reducing the compressor power. A simulation model is adapted and parametrized by the experimental data. Based on this model the performance is evaluated.

 The open access of the operational data is much appreciated, as well as the indication of the sensor specifications of type and uncertainty. This allows a very good reproducibility of the results.

 In terms of statistical evaluation of the data, the parity plots are very good and helpful. Some performance indicators might be helpful to compare especially the obtained improvements with the improved correlation.

 In general, the publication is very well written and structured. In order to improve the quality of the paper even further, I recommend addressing the following comments:

1. Some minor typos and wordings should be corrected by a final proofreading (i.e. relative literature -> related literature, cubed exponent -> inverse cubed exponent, heat pumps systems -> heat pump systems, liluratre -> literature, regulation -> control, Wwhere -> Where, chocking valve -> choking valve,  …)
2. The quality and resolution of some of the figures need to be improved.
3. Is there any benefit in introducing weighting factors in the minimization function (equation 1)?
4. The authors note that data from the manufacturer and from experiments have been made. Is there any differentiation made between the two data sets? Or have they been merged? Has a separate analysis been performed?
5. Caption of Figure 1: To be more precise, please introduce dS=0 isentropic as well as dV=0 isochoric.
6. For the isentropic efficiency, I recommend to use \eta instead of n_is (page 6).
7. Please comment on the “best-fit procedure”. Out of which different correlations has been chosen?
8. Figure 3 could be displayed in log-formatted y-axis to allow for a better differentiation.
9. For the temperature ranges of the test conditions on page 10: Please indicate the temperature steps, if possible.
10. The authors write: “The heat pump was operating many hours per day”, but what was determining the operation: Experiments or daily ambient temperature? Maybe the authors can add a short explanation.
11. It is not clear how the authors determined steady-state for the operational points. It would be good to add a short explanation.
12. Is the compressor/heat pump cycle operated with pure working fluid or has a lubrication oil been added?
13. In the uncertainty analysis a standard reference on error propagation can be added.
14. Tables 5-6: Please harmonize the accuracy of parameter and uncertainty in terms of digits.
15. Fig. 7-9: Why is there no operational point at T_ev,in=10 °C and T_w,cond,out=35 °C?
16. With respect to the results section: A direct comparison between the two fluids would be helpful to facilitate the discussion of the results.
17. More importantly, I am missing a comparison of the working fluid and its thermo-physical parameters (i.e. supported by T-s-diagram). This will facilitate the explanations in the deviations of pressure drop and performance between the two fluids.

Is the study design appropriate and does the work have academic merit?

Yes

Is the work clearly and accurately presented and does it cite the current literature?

Yes

If applicable, is the statistical analysis and its interpretation appropriate?

Partly

Are all the source data underlying the results available to ensure full reproducibility?

Yes

Are the conclusions drawn adequately supported by the results?

Yes

Are sufficient details of methods and analysis provided to allow replication by others?

Yes

Reviewer Expertise:

Organic Rankine cycle power systems with expertise in both, experimental and numerical.

I confirm that I have read this submission and believe that I have an appropriate level of expertise to confirm that it is of an acceptable scientific standard, however I have significant reservations, as outlined above.

# Response

**Published:** 2022-11-10

**Authors:** George Kosmadakis

**Affiliations:** National Center for Scientific Research Demokritos, Greece

**Reviewer**: A scroll compressor designed for R407C has been investigated for R1234ze(E). The main feature of the compressor is its capability of vapor injection in between the compression process. This increases the efficiency by reducing the compressor power. A simulation model is adapted and parametrized by the experimental data. Based on this model the performance is evaluated.

 The open access of the operational data is much appreciated, as well as the indication of the sensor specifications of type and uncertainty. This allows a very good reproducibility of the results.

***Authors**: We would like to thank the Reviewer for his comments. His suggestions, time and effort to review this work are really appreciated.*

 In terms of statistical evaluation of the data, the parity plots are very good and helpful. Some performance indicators might be helpful to compare especially the obtained improvements with the improved correlation.

***Authors**: In order to compare the improved semi empirical model with the standard one, Table 9 includes some performance indicators concerning minimum, maximum and average deviation of the predicted variables with R407C refrigerant. A similar comparison is included in Table 11 for R1234ze(E).*

**Reviewer**: In general, the publication is very well written and structured. In order to improve the quality of the paper even further, I recommend addressing the following comments: Some minor typos and wordings should be corrected by a final proofreading (i.e. relative literature -> related literature, cubed exponent -> inverse cubed exponent, heat pumps systems -> heat pump systems, liluratre -> literature, regulation -> control, Wwhere -> Where, chocking valve -> choking valve…)

***Authors**: Thank you for your comments. All the typographical errors and wordings have been corrected.*

**Reviewer**: The quality and resolution of some of the figures need to be improved.

***Authors*** *: All the figures will be uploaded again with >= 300 dpi resolution.*

 Is there any benefit in introducing weighting factors in the minimization function (equation 1)?  

***Authors**: According to the literature, compressor or expander semi-empirical models are usually calibrated with no weighting factor in the minimization function, in order to favor evenly each factor (Temperature, mass flow rate and electricity). This has been also followed here.*

**Reviewer**: The authors note that data from the manufacturer and from experiments have been made. Is there any differentiation made between the two data sets? Or have they been merged? Has a separate analysis been performed?

***Authors**: The compressor data with R407C are extracted from the manufacturer selection software. These data then have been used, in order to calibrate the semi empirical model and to compare the outputs of the model with the manufacturer’s results for validation reasons. The other data set was a separate one, and was obtained from the actual testing of the heat pump unit at the lab with the same compressor as for R407C but with R1234ze(E) refrigerant. These two datasets were analyzed and processed separately but they have been used as inputs to the same semi-empirical model.*

**Reviewer**: Caption of Figure 1: To be more precise, please introduce dS=0 isentropic as well as dV=0 isochoric.

***Authors**: The suggested changes have been made.*

**Reviewer**: For the isentropic efficiency, I recommend to use \eta instead of n_is (page 6).

***Authors*** *: The symbol has been corrected. * 

**Reviewer**: Please comment on the “best-fit procedure”. Out of which different correlations has been chosen?

***Authors**: The fitting has been made based on the Zigrang & Sylvester correlation. This is now commented in the text as well.*

**Reviewer**: Figure 3 could be displayed in log-formatted y-axis to allow for a better differentiation.

***Authors**: The x axis of Figure 4 (Previous Figure 3) has been formatted to logarithmic in order for the trend to be more “linear”. Logarithmic form of the y axis brings a minor improvement. * 

**Reviewer**: For the temperature ranges of the test conditions on page 10: Please indicate the temperature steps, if possible.

***Authors**: The temperature steps (setpoints) at the evaporator were three (10, 15 and 25 ^o^C). At each setpoint in the evaporator, the heat pump operated to heat up a large tank, with the condenser temperature started from about 30 ^o^C and gradually reached 50 ^ο^C.*

**Reviewer**: The authors write: “The heat pump was operating many hours per day”, but what was determining the operation: Experiments or daily ambient temperature? Maybe the authors can add a short explanation.

***Authors**: The operation was only for experimental reasons. The evaporator and condenser water side were connected to two large tanks. The “cold” tank at the evaporator side was equipped with electric resistances while the one at the condenser with an air source heat pump. Therefore, the temperature could be controlled in both tanks. This is included in the text just before the new Fig. 6.*

**Reviewer**: It is not clear how the authors determined steady-state for the operational points. It would be good to add a short explanation.

***Authors**: For the operational points and the final data set obtained from the measurements, 10-minute averaged values are selected from different working conditions. A short explanation has been added just before the new Table 5.*

**Reviewer**: Is the compressor/heat pump cycle operated with pure working fluid or has a lubrication oil been added?

***Authors**: The compressor is hermetic with an internal oil lubrication system which is achieved by pumping the oil through a haft from a sump located at the bottom part of the compressor. There is no additional (external) lubrication.*

**Reviewer**: In the uncertainty analysis a standard reference on error propagation can be added.

***Authors**: A standard reference has been added: Taylor B N and Kuyatt C E 1994 Guidelines for Evaluating and Expressing the Uncertainty of NIST Measurement Results (Gaithersburg, MD: National Institute of Standards and Technology) NIST Technical Note 1297.*

**Reviewer**: Tables 5-6: Please harmonize the accuracy of parameter and uncertainty in terms of digits.

***Authors**: Done.*

**Reviewer**: Fig. 7-9: Why is there no operational point at T_ev,in=10 °C and T_w,cond,out=35 °C?

***Authors**: Unfortunately, there were no available experimental data sets for this combination of temperatures. * 

**Reviewer**: With respect to the results section: A direct comparison between the two fluids would be helpful to facilitate the discussion of the results.

***Authors**: A comparison between the two fluids has been added regarding the performance parameters with the two refrigerants in the results section.*

**Reviewer**: More importantly, I am missing a comparison of the working fluid and its thermo-physical parameters (i.e. supported by T-s-diagram). This will facilitate the explanations in the deviations of pressure drop and performance between the two fluids.

***Authors**: We would like to thank the reviewer for pointing this out. A comparison between the two fluids in terms of thermo-physical properties has been added in the testing facilities section.*

# Peer review: Reviewer response for version 1

**DOI:** 10.21956/openreseurope.15834.r29592

**Published:** 2022-07-08

**Authors:** Nicolas Tauveron

**Affiliations:** 1 CEA, Liten, Univ., Grenoble Alpes University, Le Bourget du Lac, France

The article deals with a very interesting subject: an improved heat-pump, including new "clean" (GWP<10) fluid. The paper concentrates on one of the main component of such system: the compressor. The compressor is of scroll technology. The scientific contribution is rather complete, involving experimental data and numerical model.

 From a general point of view, the paper is clear, well-written and easy to follow.

 The introductory part offers an interesting presentation of the context. However, I suggest that the auhtors include a precise definition of high-temperature heat pump (HTHP) and low GWP refrigerant. Moreover, some additional elements concerning lubrication and oil could be useful in the presentation.

 The second part is devoted to the model presentation and is generally clear, except the Wcp definition. It is not clear to me if this value includes mechanical and electrical conversion or not.

 Next section concerns the experimental test rig. The fact that the design fluid was R407C (and not R1234zd(E)) could be commented through a table or a P-h diagram. The facility is well described, but a general thermodynamic diagram in complement to Figure 5 could help the reader. Specific comments about the use of two types of expansion valve could be interesting.

 Next step concerns cycle analysis. We suppose that heat losses were not considered during cycle analysis (excepting in the compressor); perhaps we miss it, otherwise this could be clarified.

 Next section concerns a technical discussion on the results. Two main technical elements are underlined and discussed. The first concerns the question of pressure losses increase in the case of using R1234zd(E). The technical reasons are well identified: a decrease of Re leading to quasi-laminar regime. The authors investigate more accurate correlations & models to take into account flow regime modification. These elements are clear and convincing. Perhaps the authors could suggest some geometrical modifications to increase fluid velocity or turbulence.

 The second concerns "compressor losses" and is, to my opinion, the most debatable part of the work. We clearly understand that the models are used to determine parameters (Table 7 for R407C and Table 9 for R1234zd(E)). Compressor losses increase is coherent with the parameters found. However, it is really surprising that some parameters are highly modified, such as the different AU, "constant terms for losses", etc ... values between the two fluid cases. I suggest that the authors could explain the main modifications due to fluid properties as they did for pressure losses modification. An example concerns AUam value modification from 12 to 1600: A(=area) should not vary a lot, and U can certainly vary a lot due to regime modification and consequently Nusselt value. A short analysis of Nusselt correlations could help the reader to understand some effects. The other example concerns the 'constant term' of electrical pressure losses: could the authors indicate why this 'constant' varies between the two configurations (276->490). I would suggest, but I do not know if it is really possible, that the approach could alternatively consider some parameters kept constant between the two configurations and the others would vary. Such an approach could help to give more predictive (and not only descriptive) results.

 The conclusion is clear, honest and really useful for the reader as the different points of the work are presented. I have appreciated that they are quite nuanced.

Is the study design appropriate and does the work have academic merit?

Yes

Is the work clearly and accurately presented and does it cite the current literature?

Yes

If applicable, is the statistical analysis and its interpretation appropriate?

Yes

Are all the source data underlying the results available to ensure full reproducibility?

Yes

Are the conclusions drawn adequately supported by the results?

Yes

Are sufficient details of methods and analysis provided to allow replication by others?

Yes

Reviewer Expertise:

Applied thermodynamic

I confirm that I have read this submission and believe that I have an appropriate level of expertise to confirm that it is of an acceptable scientific standard.

# Response

**Published:** 2022-11-10

**Authors:** George Kosmadakis

**Affiliations:** National Center for Scientific Research Demokritos, Greece

**Reviewer**: The article deals with a very interesting subject: an improved heat-pump, including new "clean" (GWP<10) fluid. The paper concentrates on one of the main component of such system: the compressor. The compressor is of scroll technology. The scientific contribution is rather complete, involving experimental data and numerical model. From a general point of view, the paper is clear, well-written and easy to follow.

***Authors**: We would like to thank the Reviewer for his valuable comments which greatly help to improve this work.*

**Reviewer**: The introductory part offers an interesting presentation of the context. However, I suggest that the authors include a precise definition of high-temperature heat pump (HTHP) and low GWP refrigerant. Moreover, some additional elements concerning lubrication and oil could be useful in the presentation.

***Authors**: A more precise definition according to literature has been added in the introduction for both low GWP refrigerants and HTHPs according to their typical operating temperatures. In addition, some comments have been provided concerning the increase of oil performance when a compressor with an economizer port is preferred (due to the lower temperatures).*

**Reviewer**: The second part is devoted to the model presentation and is generally clear, except the Wcp definition. It is not clear to me if this value includes mechanical and electrical conversion or not.

***Authors**: The compressor electrical power Wcp  includes electromechanical conversion and all related losses. To make this clearer, its definition has been enriched just after Eq. (7) in the revised text. * 

**Reviewer**: Next section concerns the experimental test rig. The fact that the design fluid was R407C (and not R1234zd(E)) could be commented through a table or a P-h diagram. The facility is well described, but a general thermodynamic diagram in complement to Figure 5 could help the reader. Specific comments about the use of two types of expansion valve could be interesting.

***Authors**: The two refrigerants R407C and R1234ze(E) are now compared in a new section, including a table with their thermo-physical properties (new Table 3) and a figure with the compressor envelope for each refrigerant. Additionally, some comments for the operation and control of the two expansion valves have been included. Furthermore, in Figure 6 (old figure 5) the P-H diagram of the thermodynamic cycle has been added.*

**Reviewer**: Next step concerns cycle analysis. We suppose that heat losses were not considered during cycle analysis (excepting in the compressor); perhaps we miss it, otherwise this could be clarified.  

***Authors**: The heat losses at the pipes and the pressure drop have been included in the cycle analysis, in order to calculate the fluid properties at the inlet of each component (e.g. plate heat exchangers, compressor, expansion valve, etc.). A short explanatory comment has been added in the revised text in the Data reduction section (before Table 6) to make this clearer.*

**Reviewer**: Next section concerns a technical discussion on the results. Two main technical elements are underlined and discussed. The first concerns the question of pressure losses increase in the case of using R1234zd(E). The technical reasons are well identified: a decrease of Re leading to quasi-laminar regime. The authors investigate more accurate correlations & models to take into account flow regime modification. These elements are clear and convincing. Perhaps the authors could suggest some geometrical modifications to increase fluid velocity or turbulence.

***Authors**: The compressor is a commercial one and for that reason the suction port has a fixed geometry with a specific diameter. Therefore, no modifications could be applied, with the only option to replace the compressor with a compatible one with R1234ze(E), but without an economizer port.*

**Reviewer**: The second concerns "compressor losses" and is, to my opinion, the most debatable part of the work. We clearly understand that the models are used to determine parameters (Table 7 for R407C and Table 9 for R1234zd(E)). Compressor losses increase is coherent with the parameters found. However, it is really surprising that some parameters are highly modified, such as the different AU, "constant terms for losses", etc ... values between the two fluid cases. I suggest that the authors could explain the main modifications due to fluid properties as they did for pressure losses modification. An example concerns AUam value modification from 12 to 1600: A(=area) should not vary a lot, and U can certainly vary a lot due to regime modification and consequently Nusselt value. A short analysis of Nusselt correlations could help the reader to understand some effects. The other example concerns the 'constant term' of electrical pressure losses: could the authors indicate why this 'constant' varies between the two configurations (276->490). I would suggest, but I do not know if it is really possible, that the approach could alternatively consider some parameters kept constant between the two configurations and the others would vary. Such an approach could help to give more predictive (and not only descriptive) results.

***Authors**: The semi-empirical model which is developed and used includes the main losses but cannot predict all the phenomena especially when the compressor works near to the envelope limits. For example, AUamb is related to the shell temperature of the compressor. This high value in the case of R1234ze(E) has more mathematical than physical meaning and states that the shell temperature needs to be reduced so that it transfers more heat to the surroundings, in order to avoid heating up the refrigerant at the suction, trying to match with the experimental measurements. Due to the high pressure loss at the suction, the compression starts from lower temperature and ends to lower discharge temperature accordingly. To possibly resolve this un-physical values, the shell envelope could be split in two regions, one for the upper part (the hotter) and the other for the bottom (the colder), but in this case the complexity increases, because two shell temperatures need to be considered. This is to be evaluated in the future, whether it could contribute to the model accuracy. A short comment has been added in the revised text (2 ^nd^ paragraph after new Table 10) to clarify this. Moreover, the parameters such as Vs,cp, Κ, αcp, and Ẇloss,0 are expected not to vary for different fluids, since they don’t depend on refrigerant properties. However, the semi-empirical model accuracy is enhanced when these parameters are recalculated during the calibration process for every refrigerant. This could be related to the assumptions of the model as well as the systematic and random errors of the data set used for the calibration.*

**Reviewer**: The conclusion is clear, honest and really useful for the reader as the different points of the work are presented. I have appreciated that they are quite nuanced.

***Authors**: We would like to thank you once again for your valuable comments, suggestions and your precious time spent in reviewing our manuscript.*
